# The dynamic proliferation of CanSINEs mirrors the complex evolution of Feliforms

**DOI:** 10.1186/1471-2148-14-137

**Published:** 2014-06-20

**Authors:** Kathryn B Walters-Conte, Diana LE Johnson, Warren E Johnson, Stephen J O’Brien, Jill Pecon-Slattery

**Affiliations:** 1 Department of Biology, American University, 101 Hurst Hall 4440 Massachusetts Ave, Washington, DC 20016, USA; 2 Department of Biological Sciences, The George Washington University, 2036 G St, Washington, DC 20009, USA; 3 Smithsonian Conservation Biology Institute, National Zoological Park, Front Royal, VA 22630, USA; 4 Dobzhansky Center for Genome Bioinformatics, St. Petersburg State University, 41 A, Sredniy Avenue St., Petersburg 199034, Russia

**Keywords:** Incomplete lineage sorting, SINEs, Carnivora, Speciation, transposable elements, Adaptation, Feliformia, Felidae

## Abstract

**Background:**

Repetitive short interspersed elements (SINEs) are retrotransposons ubiquitous in mammalian genomes and are highly informative markers to identify species and phylogenetic associations. Of these, SINEs unique to the order Carnivora (CanSINEs) yield novel insights on genome evolution in domestic dogs and cats, but less is known about their role in related carnivores. In particular, genome-wide assessment of CanSINE evolution has yet to be completed across the Feliformia (cat-like) suborder of Carnivora. Within Feliformia, the cat family Felidae is composed of 37 species and numerous subspecies organized into eight monophyletic lineages that likely arose 10 million years ago. Using the Felidae family as a reference phylogeny, along with representative taxa from other families of Feliformia, the origin, proliferation and evolution of CanSINEs within the suborder were assessed.

**Results:**

We identified 93 novel intergenic CanSINE loci in Feliformia. Sequence analyses separated Feliform CanSINEs into two subfamilies, each characterized by distinct RNA polymerase binding motifs and phylogenetic associations. Subfamily I CanSINEs arose early within Feliformia but are no longer under active proliferation. Subfamily II loci are more recent, exclusive to Felidae and show evidence for adaptation to extant RNA polymerase activity. Further, presence/absence distributions of CanSINE loci are largely congruent with taxonomic expectations within Feliformia and the less resolved nodes in the Felidae reference phylogeny present equally ambiguous CanSINE data. SINEs are thought to be nearly impervious to excision from the genome. However, we observed a nearly complete excision of a CanSINEs locus in puma (*Puma concolor*). In addition, we found that CanSINE proliferation in Felidae frequently targeted existing CanSINE loci for insertion sites, resulting in tandem arrays.

**Conclusions:**

We demonstrate the existence of at least two SINE families within the Feliformia suborder, one of which is actively involved in insertional mutagenesis. We find SINEs are powerful markers of speciation and conclude that the few inconsistencies with expected patterns of speciation likely represent incomplete lineage sorting, species hybridization and SINE-mediated genome rearrangement.

## Background

Repetitive short interspersed elements (SINEs) are ubiquitous eukaryotic retrotransposons. SINE sequences are approximately 70–700 base pairs (bp) averaging about 250 bp [1] with most organized into an RNA gene-derived region, a di-nucleotide repeat region and terminating in a poly A or poly A/T tail [2-4]. SINEs are “non-autonomous” such that amplification and integration is dependent on enzymes derived from the host genome and long interspersed nuclear elements (LINEs) [5]. Proliferation is initiated via recognition of promoter boxes residing in the tRNA-related region of the genomic “master-copy” by host-derived RNA polymerase III and eventually results in novel retrotransposed copies [6]. SINEs constitute roughly 10% of the mammalian genome [1,7-10] and classification into family or subfamily designations is based on sequence variation and presence in specific evolutionary lineages [5,9,11,1,14].

Initially viewed as “junk” DNA without function, seminal studies in rodents [15,16] and primates [17-19] indicate a far more important role for SINEs in genome organization, gene evolution, and disease. For example, germ-line insertions are correlated with non-homologous genome rearrangements, generation of novel coding sequences, alteration of regulatory elements and are linked with the origin and evolution of highly conserved non-coding elements in mammals [18,20-26]. Within somatic cells, *de novo* SINE integration can disrupt pathways involved with cell differentiation [27], modulate intracellular targeting of mRNAs [15] and potentially provide other cell-specific phenotypes [28].

Direct phenotypic variation is possible by altering gene expression via insertion into coding regions or interference from the internal RNA polymerase promoters in SINEs [29]. Analysis of the dog genome revealed SINE insertion polymorphisms resulting in anti-sense transcription that provide alternate splice site junctions [30]. For example, alterations of fur color [31], muscular disorders [32,33] and body size diversity [34,35] in Canidae are correlated with SINE insertions associated respectively with *SILV, PTPLA* and *IGF1*. In addition, SINE insertion into an exon of *STK38L* causes retinal degeneration [36] and an ancient SINE locus serves as an enhancer for fibroblast growth factor 8 (*Fgf8*) during mammalian brain formation [37].

SINEs are highly informative markers used in mammalian phylogenetic and population genetic studies of cetaceans [38], carnivores [39-42], primates [11,43,44], rodents [45,46], xenarthrans [47], marsupials [48,49] and the diverse assemblage of African species termed Afrotheria [50]. With few mechanisms for precise removal, SINE insertions are nearly homoplasy-free unidirectional markers and therefore informative in deciphering complex patterns of speciation [40,50-52]. In general, phylogenetic inferences rely upon presence and absence data of SINE loci among taxa. However, instances of parallel insertions unrelated to phylogenetic associations have been detected through sequence data [41,44,53,54] and indicate SINEs target specific sequence motifs during proliferation [1,55-57]. Incomplete lineage sorting of ancestral polymorphisms via ongoing hybridization or introgression among populations may cause contradictory findings in SINE-based phylogenetic reconstructions [58,59]. Consequently, accurate species trees are required to serve as a reference phylogeny for interpreting patterns of insertion and sequence divergence at SINE loci.

Here we utilized the well-resolved phylogeny of Felidae as a species tree to investigate the evolution a lesser known family of mammalian SINEs; those within the Order Carnivora, termed CanSINEs. The two suborders of Carnivora are Caniformia (dog-like) and Feliformia (cat-like). Caniformia is organized into Ursidae (bear), Canidae (domestic dogs, wolves, foxes, jackals, coyotes), Otariidae (eared seals), Odobenidae (walrus), Phocidae (earless seals), Mustelidae (badgers, weasels and otters), Mephitidae (skunks), Procyonidae (raccoons, coatis, kinkajous, olingos, ringtails and cacomistles), Ailuridae (red panda) [60,61]. Feliformia is composed of Felidae (cats), Viverridae (civets, genets, African linsang), Prionodontidae (Asiatic linsang), Eupleridae (Malagazy carnivores), Naniniidae (african palm civet), Herpestidae (mongooses), and Hyaenidae (hyenas) [60]. Initially discovered in multiple species of Caniformia [3,62,63], CanSINEs were presumed absent from Feliformia. This was revised upon further studies of the feline Y-chromosome [53,64] and through whole genome sequence analyses [8,65].

We used comparative methods to sequence CanSINEs within Feliformia with specific focus on the Felidae. Thirty-seven cat species augmented by representatives from related Feliform represent roughly 44 million years (MY) of divergence (see Additional file 1: Table S1) [40]. The extant cat species diverged into eight lineages in a nearly starburst pattern over 10 MY [66], and have largely maintained synteny in chromosome architecture [10]. Roughly 10-11% of a felid genome is comprised of SINEs [10]. We identified 93 new CanSINE loci, which were divided into quiescent and active subfamilies. In addition, we found empirical evidence of the effects of rapid speciation and imprecise SINE excision on phylogenetic consistency.

## Results

We applied both *in silico* genome mining and PCR-based approaches to identify feliform CanSINE loci, which were then sequenced in 37 extant Felidae species and five additional representatives from Prionodontidae, Viverridae, Herpestidae, and Hyaenidae. First, direct *in silico* genome annotation of the domestic cat (*F. catus*), verified against the dog genome (*Canis familiaris*), identified 29 new CanSINE loci (see Additional file 2: Table S2A). Second, a SINE-to-SINE PCR method [67] isolated another 30 SINE-flanked genomic regions in exotic felids (see Additional file 2: Table S2B). Among the 59 total amplified regions, 21 (35%) included two or more independent insertions in Feliformia species. Together these represent 93 previously uncharacterized CanSINE loci (Additional file 3: Tables S3 and Additional file 4: Table S4).

### CanSINE insertion hotspots

**Figure 1:**
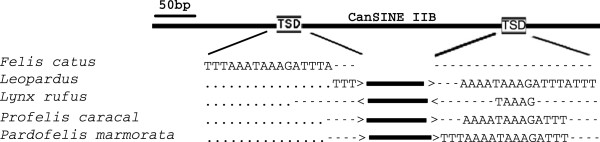
**CanSINE insertions at locus 133135.** Arrangement of 4 unique CanSINE insertion events occurring at locus 133135 in the caracal (*Profelis caracal*), marbled cat (*Pardofelis marmorata*), ocelot lineage (genus: *Leopardus*) and bobcat (*Lynx rufus*) with the homologous *F. catus* sequence as a reference. The *L. rufus* SINE is in reverse orientation. The independent insertions have overlapping target site duplications (boxed).

**Figure 2:**
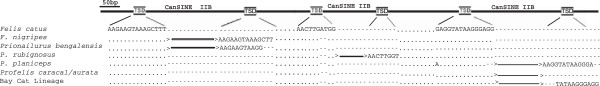
**CanSINE insertions at locus 212075.** Arrangement of six unique insertion events occurring at locus 212075 in the Asian Leopard Cat lineage species of rusty spotted cat (*P. rubiginosus*), flat-headed cat (*P. planiceps*), Asian leopard cat (*P. bengalensis*), and black-footed cat (*F. nigripes*) along with synapomorphies of the African golden cat clade (*P. caracal/aurata*), and the Bay Cat lineage. The insertions in the Asian Leopard Cat lineage species (*P. bengalensis* N = 9) and flat-headed cat (*P. planiceps* N = 5) are unfixed.

CanSINEs from different lineages targeted homologous loci during proliferation and retrotransposition within the genome. At least three inserts were found in 8 of the 21 multiple-insert loci (62%) in Felidae (See Additional file 5: Table S4). For example, inserts at locus 133135 occurred in unrelated *Lynx rufus*, *Profelis caracal* and *Pardofelis marmorata*, along with a synapomorphic insertion shared in the seven species of the ocelot lineage (Figure 1, see Additional file 5: Figure S1, see Additional file 4: Table S4). Each of these four CanSINEs was flanked by species-specific, overlapping target site duplication sequence (TSD). Independence of the four insertion events is verified by multiple nucleotide indels in the microsatellite and poly A/T segments. Furthermore, the *L. rufus* SINE is in the reverse orientation. Similarly, locus 212075 contained six independent insertion events including: 1) a shared synapomorphy defining the bay cat lineage, 2) a shared synapomorphy of *P. caracal*/*P. aurata* and 3) autapomorphic insertions in *Felis nigripes*, *P. rubiginosus, P. bengalensis* and *P. planiceps*. In the latter case, insertions in *P. bengalensis* (n = 9) and *P. planiceps* (n = 7) were unfixed (Figure 2, see Additional file 6: Figure S2, see Additional file 4: Table S4,).

An examination of patterns of sequence divergence of both tRNA and genomic flanking regions suggests the insertions at 212075 occurred independently among species. In *F. nigripes* and *P. bengalensis* CanSINEs were flanked by different TSDs and the percent identity was 81.6% within the SINE regions compared to 96.4% in the regions flanking the SINE (Figure 2, see Additional file 6: Figure S2, see Additional file 2: Table S2). Similarly, *P. planiceps* and the bay cat lineage CanSINEs are flanked by different, but overlapping, TSDs and the percent identity was 82.8% within SINE regions compared to 97.2% in the 126 nucleotides flanking the SINE. While these sequence diversity estimates do not definitively preclude post insertion mutations, they are consistent with independent retrotransposition events by unique RNA templates*.*

### Evolutionary assessment of feliform CanSINEs

**Figure 3:**
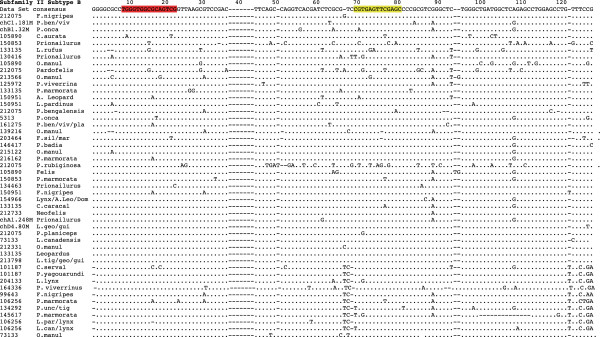
**Alignment of tRNA-related SINE subregions found among Feliformia.** Diagnostic indels are attributed to SINE subfamily and subtype distinctions. The RNA polymerase III boxes are indicated in red (A) and yellow (B).

**Figure 4:**
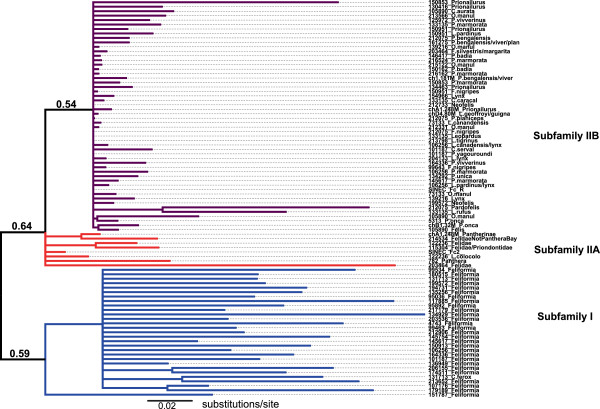
**Phylogeny of feliform CanSINEs.** A consensus phylogeny based on neighbor-joining optimization of feliform CanSINEs, based on 87 aligned tRNA-related regions and 2 RepBase voucher sequences, depicts two SINE subfamilies, I and II, with internal clades of subtypes A and B in subfamily II. Numbers in bold indicate support scores based on 1000 pseudo-replicates. Branch colors blue, red and violet indicate sequences characterized belonging to subfamily I, subfamily II type A and subfamily II type B, respectively. The full alignment including terminal labels is in Figure 3.

**Figure 5:**
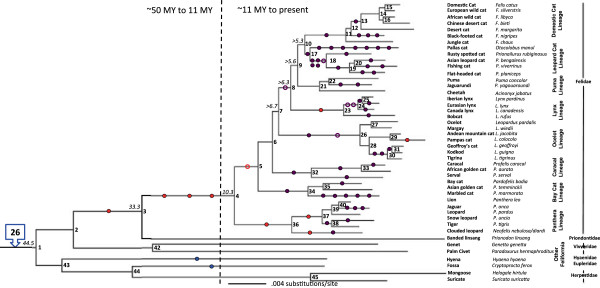
**Distribution of CanSINE instances within the feliform/felid phylogeny.** Ninety-three CanSINE insertion events are mapped onto a maximum-likelihood Feliformia/Felidae species tree reproduced using ~18 kbp of nuclear DNA from Johnson et al. [66]. Twenty-six subfamily I insertion events occurring in the feliform ancestor are indicated by the blue arrow box. Blue circles indicate 2 additional subfamily I CanSINEs in non-Felidae feliforms. Red and violet circles indicate more recent subfamily II type A and subfamily II type B insertions respectively. Solid circles denote fixed insertions present in all species within a lineage. Open circles denote unfixed insertions within a lineage (i.e. not all species in the lineage as presented have the insert). Approximate divergence times for discussed nodes are noted.

Based on alignment and phylogenetic reconstruction of conserved tRNA regions, we identified major CanSINE lineages defined by distinct motifs, which we have designated as subfamilies and subtypes (Figures 3 and 4). Subfamily I members share a diagnostic ‘TCCTGAT’ motif at position 36 within the 5’ tRNA-related region. Additional variants within the tRNA-related region of ‘CA’ or ‘GT at position 116 and ‘GGGA’ or AAGA’ at position 138 were diagnostic for subtypes IA and IB respectively (Figure 3). Loci in subfamily II share a ‘GGCTCGG’ motif at position 118 within the tRNA region and subtypes IIA and IIB are delineated by an insertion/deletion (‘T’) at position 51 within the 5’ tRNA-related region (Figure 3). Notably, there is an A > T polymorphism at position 70 within the RNA polymerase B box that segregates nearly perfectly with subfamilies I and II and a T > G polymorphism in the RNA polymerase A box that is specific to subtype IIB (Figure 3). In addition, published SINE voucher sequences annotated from *F. catus* clustered within the two subtypes of subfamily II [i.e. SINEC_Fc1 grouped with subtype IIB and SINEC_Fc2 grouped with subtype IIA (Figure 4)].Phylogenetic differences between the two subfamilies are concordant with ancestral versus recent nodes within the Feliformia species tree. The most ancestral CanSINE lineage is subfamily I, composed of loci conserved among the 6 families in Feliformia and thus likely arose in a common ancestor ~50 MYA (Figure 5). Within subfamily II, four of the 10 subtype IIA CanSINEs arose ~35 MYA in a common ancestor of Felidae and Priondontidae, in the progenitor of Felidae or early in the initial Felidae radiation, while the remaining six are scattered among more recent lineages (Figure 4). In contrast, all 49 of subtype IIB insertions are localized to individual felid clades or are unique to a single species and thus likely arose within the last 5 million years (Figure 5).

If CanSINE proliferation and subsequent sequence divergence is correlated with evolutionary time, then more ancient inserts will have greater nucleotide variation then those of recent origin. The more ancestral subfamily I is three times more diverse (0.298 substitutions/site) than subfamily II (0.090 substitutions/site) (Table 1). The most variable CanSINE was subtype IA (0.271 substitutions/site) and the least variable was subtype IIB (0.068 substitutions/site) (Table 1). These results suggest that measures of average sequence divergence observed in CanSINE lineages, calibrated by the feliform phylogeny, are estimates of time since periods of active proliferation.

**Table 1:** Mean genetic distance (substitutions/site) of feliform SINE subfamilies and subtypes with standard deviation

**SINE type**	**Genetic distance**	**SD**
All SINEs	0.205	0.133
Subfamily I	0.298	0.083
Subfamily II	0.090	0.068
Subtype IA	0.271	0.079
Subtype IB	0.189	0.045
Subtype IIA	0.137	0.066
Subtype IIB	0.068	0.052

Using an unpaired T-test, differences in the mean genetic distances between each subfamily and subtypes within each family were significant with p <0.0001.

### CanSINE evolution in the cat family Felidae

The phylogenetic fidelity of the 93 CanSINE loci varied among the hierarchical nodes within Feliformia. The 33 species-specific loci were distributed among the eight major Felidae lineages. An additional 26 CanSINEs supported the monophyly of Feliformia (Figure 5: node 1). Another three insertions supported the monophyly of Felidae and one CanSINE locus supported the sister group relationship between Felidae and Priondontidae (Figure 5: nodes 3 and 4). Two unique insertions were found in non-Felidae representatives of *H. hyaena* and *C. ferox.* Twenty CanSINE loci were diagnostic for internal clades within Felidae while seven of the eight major felid lineages had diagnostic loci (Figure 5: nodes 36, 34, 32, 26, 23, 17 and 11 respectively). Intra-lineage markers included loci defining clades within the panthera, Asian leopard cat, caracal, ocelot and felis lineages (Figure 5: nodes 13, 18, 19, 30, 31, 33, 37 and 38).

### Discordant phylogenetic inferences correlate with polymorphic loci

**Figure 6:**
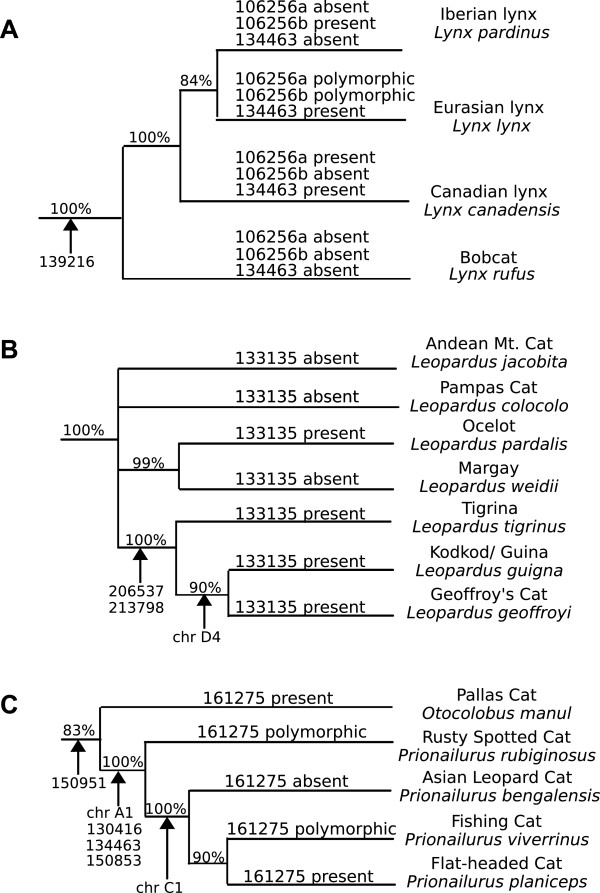
**CanSINE insertion sites incongruent with prior phylogenic analyses.** The model topologies shown are based on maximum-likelihood reconstruction using 18,853 bp of nuclear DNA, with bootstrap scores noted to the left of each node and divergence time estimates between nodes in gray (Johnson et al 2006) [66]. **A)** An insertion at locus 106256 is present in *L. canadensis* (N=22), polymorphic in *L. lynx* (N=23) and absent in *L. pardinus* (N=8), while another insertion near the same site is absent from *L. canadensis*, polymorphic in *L. lynx* and present in *L. pardinus*. A third insertion at locus 134463 is present in all *L. canadensis* and *L. lynx* and absent in all *L. pardinus*. **B)** An insertion at locus 133135 has a paraphyletic distribution among the ocelot lineage species; *Leopardus pardalis* (N=10), *L. jacobita* (N=2), *L. tigrina* (N=9), *L. guigna* (N=3) and *L. geoffroyi* (N=11), *L. wiedii* (margay, N=6) and L. *colocolo* (pampas cat, N=3). The placement of *L. jacobita* and *L. colocolo* with respect to the remaining *Leopardus* species has yet to be determined with statistical confidence and thus is depicted here as a polytomy. **C)** An insertion at locus 161275 is present in all *P. viverrinus* (N=7), absent in *P. planiceps* (N=5) and polymorphic among *P. bengalensis* (N=9) and *P. rubiginosus* (N=2). However, 4 other SINE insertion sites support the monophyly of *Prionailurus* and one insertion supports the monophyly of *P. bengalensis*, *P. viverrinus* and *P. planiceps*.

The 93 CanSINE loci presented here were mapped to a phylogeny based on multiple optimality criterion described by Johnson et al. (Figure 5) [66]. However, alternate branch topologies and phylogenetic ambiguities are indicated by six of the 93 CanSINE loci. In the lynx lineage, an orthologous insertion at locus 106256 was homozygous in all *L. pardinus* individuals while a second independently derived insertion, occurring 315 bp downstream of the first, was homozygous in *L. canadensis.* These orthologous insertions are polymorphic within *L. lynx*, with individuals either homozygous for one of the insertions or heterozygous, containing one copy of each (Figure 6A, see Additional file 7: Figure S3). No correlation was observed between the geographic origin of *L. lynx* individuals and CanSINE profile (see Additional file 8: Table S5). Further ambiguity of the *Lynx* genus topology was indicated by the presence of CanSINE locus 134463 in all *L. canadensis* and *L. lynx* individuals with absence from *L. pardinus* (Figure 6a, See Additional file 9: Figure S4).

Likewise, CanSINEs did not always map to expected species associations within the South American ocelot lineage. CanSINE locus 133135 is fixed in *Leopardus pardalis, L. jacobita*, *L. tigrina*, *L. guigna* and *L. geoffroyi*, yet absent in *L. wiedii* and *L. colocolo* (Figure 6B, see Additional file 10: Figure S5). In the Asian leopard cat lineage, locus 161275 is polymorphic in *P. rubiginosus* and *P. bengalensis,* fixed in *P. viverrinus* and absent from *P. planiceps* (Figure 6c, see Additional file 11: Figure S6).

To account for the possibility that the CanSINE profiles described above are the result of recent hybridization events between closely related species, mitochondrial profiles at the *NADH5* gene were obtained from all individuals representing the lynx, ocelot and Asian leopard cat lineages. We found most mtDNA haplotypes to be consistent with species designation and the previously proposed phylogenetic relationships among the Felidae species. The exception was *P. rubiginosus*, which had differing *NADH5* haplotypes identical to those found among *P. bengalensis* (see Additional file 11: Figure S6).

Two CanSINE loci that may be mapped to the “backbone” of the Felidae tree were also inconsistent with prior estimations of the initial Felidae radiation. Locus 154966 is present in all species of the domestic cat, Asian leopard cat and lynx lineages, and absent in the puma, ocelot, caracal, bay cat and panthera lineages (Figure 5: nodes 4–10, see Additional file 12: Figure S7). Locus 214534 is present in all Felidae species except those of the caracal and panthera lineages (Figure 5: nodes 4–10, see Additional file 13: Figure S8).

### Evidence for SINE Excision

**Figure 7:**
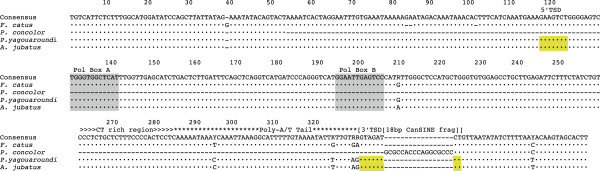
**Absence of CanSINE insertion locus 174511 in the Puma.** Loss of the target CanSINE insertion at locus 174511 and 68 adjacent nucleotides (bp 99–379) from *P. concolor* (N = 17) and replacement by an 18-nucleotide sequence (bp 81–98) similar in primary sequence to a CanSINE in the opposite orientation. Yellow-highlighted regions mark target site duplications and gray-shaded regions denote the A and B RNA polymerase III recognition sequences. The other puma lineage species, *Acinonyx jubatus* (N = 8) and *P. yagouaround* (N = 8) included the canonical CanSINE profile*.*

CanSINE locus 174511 is present in all feliform taxa with one exception. In *Puma concolor* locus 174511 includes an 18 bp reverse-oriented SINE fragment rather than a full-length SINE and mapped to 63 bp of upstream sequence (Figure 7). By contrast, the full-length SINE locus 174511 is fixed in the two puma lineage sister species, *Acinonyx jubatus* and *P. yagouaroundi.*

## Discussion

Genomic characterization of 93 novel CanSINEs in Feliformia clarifies, amends and extends existing hypotheses on SINE evolution and strongly supports the phylogenetic fidelity of these retrotransposons. In using the well-supported phylogeny of the cat family, Felidae, as a reference species tree, we provide empirical evidence for long speculated, but rarely observed, processes such as co-evolution of SINE families with the host genome, targeted insertion during CanSINE proliferation, lineage sorting of ancestral polymorphisms among closely related species, and instances of SINE excision from the genome.

### CanSINE integration targets homologous loci

The discovery of 93 CanSINE loci includes a high frequency of multiple insertions within orthologous intergenic regions. Some loci serve as apparent “hotspots” of CanSINE activity within the Felidae. For example, CanSINE locus 133135 displays four independent insertion events defined by different, yet overlapping TSDs. Likewise, locus 212075 supports six independent insertions, three of which occur in a single genus, *Prionailurus*. Similar patterns of CanSINE integration have been observed in the Caniformia suborder wherein amplification of five putative *C. familiaris* CanSINE loci revealed eight additional insertions in related species [40], and amplification of 13 intronic segments amongst caniforms revealed 26 independent insertion events [58]. Possible explanations for the likelihood of additional CanSINEs co-occurring at orthologous loci involve signature motifs associated with the L1 long interspersed element (LINE) derived endonuclease [1]. In primates, integration of SINEs (*Alu* repeats) is facilitated by the motif TTAAAA(N)_0-8_TYTNR [68]. A similar mechanism is hypothesized in whole genome assessments that found over 20% of *C. familiaris* CanSINE integration sites include a TTAAAA motif [1,69]. Likewise, CanSINE integration sites within Feliformia share similar AT-rich motifs (Tables S3 and S4) indicating target site preferences [53].

### Dynamic evolution of Feliform CanSINE lineages

Beyond the initial reporting of voucher sequences (see Repbase database http://www.girinst.org/repbase) within the domestic cat whole genome sequence [8], little is known of CanSINE evolution in Feliformia [70]. Until now, even the most current SINE resource (SINEbase, http://sines.eimb.ru) includes only one feliform specific voucher [1]. Here, phylogenetic analysis of the conserved tRNA-like regions of the 93 newly described CanSINEs reveal two distinct subfamily lineages defined by time of emergence within Felidae and further differentiated into subtypes marked by specific sequence motifs and adaptive reverse transcriptase promoter sites (Figures 3 and 4).

Subfamily I likely originated roughly 45–60 MYA when the Carnivore order first split to form two major lineages of Feliformia and Caniformia [60]. Subfamily II arose during the emergence of sister families of Prionodontidae and Felidae, with continuous diversification until present-day Felidae. The relatively smaller mean genetic distance among subfamily II CanSINEs compared to subfamily I (0.090 substitutions/site versus 0.298 substitutions/site) is consistent with subfamily II insertions being acquired more recently from either a single master copy or multiple yet similar master copies, whereas subfamily I insertions are derived from a now quiescent set of master copies and have since accumulated substitutions.

Historic and ongoing patterns of CanSINE proliferation can be inferred by both position with the feliform phylogeny and the extent of sequence divergence among loci. The more basal subfamily I is comprised of subtypes IA and IB, which each arose in a common ancestor to Feliformia (Figure 5). Significant genetic distance estimates for subtype IA and IB (Table 1) imply that each lineage may have originated from different master copies and that subtype IA may have proliferated before IB. Within subfamily II, subtype IIA master copy or copies may have had an ancient origin, inserting into a common ancestor of Felidae and Priondontidae (Figure 5). However, this subtype apparently remains a source of extant species-specific insertions as indicated by species-specific presence in *L. colocolo* and *N. neofelis* (Figure 5). Subtype IIB CanSINEs are more recent, not as genetically diverse as IIA loci, and are the source of most phylogenetically informative sites in extant Felidae (Table 1, Figure 5).

While the genetic distances among each CanSINE subfamily provide substantial evidence for a progressive evolution of CanSINEs from the Feliform ancestor to present, the phylogenetic support scores for subfamily I, subtype IIA and subtype IIB remain relatively low, 50-65%. In addition, subfamily I cannot be resolved into subtypes based on consensus of 1000 minimum evolution replicates (Figure 4). A possible explanation for this lack of resolution could be the existence of multiple master copies that can concurrently convey insertional mutagenesis, leading to the paraphyletic pattern observed in the CanSINE phylogeny. This mechanism, also known as the ‘sprout’ model, has been proposed for human *Alu*s and allows for secondary master copies to provide a minor portion of a subfamily’s members [71].

In addition, CanSINE subfamilies have distinctive polymorphisms in the pol A and pol B RNA polymerase III binding sites that may indicate adaptive evolution. As non-autonomous transposable elements, changes in host polymerase specificity can cause SINE quiescence or adaptation [72,73]. Here, the A > T mutation in polymerase box B of the recent subfamily II and the T > G transversion in polymerase box A of subfamily IIB is not observed in the more ancestral subfamily I and thus could be evidence of functional adaptation driving ongoing subfamily II proliferation (Figure 3). However, RNA polymerase III A and B boxes are known to contain degenerate sites [74] and evidence of adaptive evolution during speciation, as opposed to accumulation of random mutations, awaits further sequence analyses of RNA polymerases in Felidae.

### Deciphering CanSINE proliferation against a backdrop of rapid speciation

SINEs are generally viewed as ideal markers of genetic divergence and phylogenetic reconstruction [52,75,76]. However, inconsistencies between SINE-based results and other molecular data may occur and are tangible evidence of complex speciation events, revealing dynamic evolutionary histories. SINEs can provide an advantage over SNP-based molecular phylogenetic analyses, wherein determining inconsistency due to homoplasy (i.e. parallelism or multiple-hits) versus hemiplasy (i.e. lineage-sorting) is ambiguous [77]. Here, the Felidae reference species tree as a framework for SINE evolution is robust [66] while the few alternate topologies [42,66,78-80], provide an opportunity to test the accuracy CanSINEs as cladistic markers during rapid speciation.

Evolution of modern Felidae is marked by a nearly star-burst pattern of speciation from a common ancestor approximately 10 MYA [60]. As such, CanSINE analyses presented here reveal limitations to correct phylogenetic interpretations even at higher-order nodes within the topology. For example, the insertion at locus 154966 suggests that the lynx lineage (Figure 5: node 8) is more recently derived than the puma lineage, which is consistent with prior minimum evolution, maximum parsimony and Bayesian analysis, yet inconsistent with maximum likelihood reconstructions [66]. Similarly, the insertion at locus 214534 suggests a more basal position of the caracal lineage within Felidae rather than the bay cat lineage, while previous phylogenetic reconstructions place the bay cat lineage at a more basal position than the caracal lineage (Figure 5: node 6), with statistical support from 50-100% depending on the optimality criterion [66]. The insertion patterns at loci 154966 and 214534 can be attributed to the nearly simultaneous divergences of the lynx and puma lineages ~7 MYA and the bay cat and caracal lineages ~9 MYA [66], resulting in “ancient” incomplete lineage sorting, a phenomenon previously observed in SINE profiles of cichlid species that diverged during a similar span of time, ~5–10 MY [81].

Similarly, rapid evolution has resulted in mosaic SINE profiles that reflect complex intra-lineage speciation patterns. In the ocelot lineage, *L. jacobita* and *L. colocolo* diverged within 20,000 years from the stem lineage (Figure 5: nodes 26–27, Figure 6B), and *L. tigrinus*, *L. guigna* and *L. geoffroyi* all arose within a brief 20,000-year interval (Figure 5: nodes 30–31, Figure 6B) [66,82]. In the lynx lineage, 40,000 years separates the *L. canadensis*, *L. lynx* and *L. pardinus* species complex (Figure 5: nodes 24–25, Figure 6A). Likewise, in the Asian leopard cat lineage *P. bengalensis*, *P. viverrinus* and *P. planiceps* diverged within a 40,000-year interval (Figure 5: nodes 19–20, Figure 6C) [66,83,84]. In addition, documented instances of ongoing hybridization between species in the wild further complicate phylogenetic analyses and taxonomy [82,84,85].

These instances of rapid speciation in Felidae are correlated with incomplete lineage sorting of ancestral polymorphisms among CanSINE loci. In the lynx lineage, maximum likelihood phylogeny derived from concatenated segments of nuclear DNA indicate *L. lynx* and *L. pardinus* are sister taxa [66,83], contrary to recent Bayesian reconstructions including mitochondrial DNA [66,78] that support a more basal position of *L. pardinus* with respect to *L. lynx* and *L. canadensis*. CanSINE distributions described here reflect the nearly simultaneous and successive speciation of the lynx, a process observed repeatedly amongst mammalian lineages [59,86]. In this instance, rapid divergence resulted an ancestral polymorphism at locus 106256 becoming fixed for presence or absence in *L. canadensis* and *L. pardinus* while remaining polymorphic in *L. lynx.* In contrast, a fixed insertion at locus 134463 supports a sister taxa relationship between *L. canadensis* and *L. lynx* (Figure 6A). Additional evidence, possibly from upcoming whole-genome efforts, should reveal a more comprehensive view of lynx phylogeny [87].

Previous analyses also failed to fully resolve the phylogenetic position of *L. jacobita* and *L. colocolo* within the ocelot lineage. Depending on the molecular data types examined and the optimality criterion employed, these two species have been placed as sister taxa or as belonging to other clades within the ocelot lineage [66,82,88]. Hence, whether the presence CanSINE locus 133135 in *L. jacobita* is due to incomplete lineage sorting of a CanSINE that was present in the *Leopardus* ancestor or due to a closer evolutionary relationship between *L. jacobita* and the *L. tigrinus*, *L. geoffroyi* and *L. guigna* clade, rather than *L. colocolo*, cannot be determined (Figure 6b). Intraspecific single nucleotide polymorphisms (SNPs) present in the *L. pardinus* 133135 locus indicate the insertion was present during the genesis of this species and not inherited more recently through hybridization (see Additional file 10: Figure S5).

In some instances CanSINEs reflect ongoing and ancestral episodes of hybridization in Felidae. For example an orthologous insert at locus 161275 in *P. rubiginosus, P. bengalensis,* and *P. viverrinus* to the exclusion of *P. planiceps* is incongruent with prior strongly supported species associations and is in direct conflict with a fixed insertion site at chromosome C1 diagnostic of the *P. bengalensis*/*P. planiceps*/*P. viverrinus* clade [66,84,89] (Figure 5: nodes 18–19, Figure 6c). Notably, the two heterozygous *P. rubiginosus* CanSINE sequences differ yet are each identical to CanSINE 161275 copies in *P. bengalensis.* This in conjunction with the *P. rubiginosus NADH5* haplotype, indicates hybridization with *P. bengalensis* after the initial radiation of *Prionailurus* (see Additional file 11: Figure S6).

Further, *P. bengalensis* serves as a model of an ongoing SINE fixation process. *P. bengalensis* is divided into two putative subspecies that diverged ~2.5 MYA: a ‘northern’ population on the Asian mainland and a ‘southern’ population on the Malay Peninsula, [84,89]. The four individuals examined from the northern population are polymorphic at locus 161275, compared with four southern homozygous individuals. Albeit a small sample size, the data suggest that the populations differ in CanSINE fixation at locus 161275 and is perhaps linked with ongoing genetic drift.

Overall, our findings suggest that rapid speciation results in mosaic genomes with conflicting phylogenetic signals [43,86]. In such instances a polytomy or split network, which recognizes shared alleles between paraphyletic groups, may be a more accurate depiction of evolutionary history. As with large scale genome sequences, CanSINE data did not unequivocally resolve the Felidae into a series of bifurcating lineages, a pattern seen even in the reconstruction of basal mammalian lineages [59,90].

### SINE locus loss

Although rarely observed, perfect or near-perfect SINE excision can occur via inter or intra chromosomal recombination between insertions of the same SINE family or between flanking TSDs [9,23,21]. The excision of locus 174511 in *P. concolor*, marked by an inverted 18 bp segment, is consistent with a mechanism of non-homologous recombination. Alternatively, simple repeats that surround the insertion site may have formed a loop structure that was omitted during DNA replication (Figure 7) leading to excision. Similar evidence of SINE removal occurs in other vertebrate lineages, such as in the squamate *Darevskia* subspecies [76] and primates [23,21].

## Conclusions

The availability of whole genome sequences has dramatically increased our understanding of mammalian non-coding DNAs. By employing comparative genomics methods to identify SINE loci in domestic and exotic feliforms, two feliform-specific CanSINE subfamilies were defined based on sequence structure and taxonomic distribution. Identification of a currently active SINE subfamily with Felidae will provide opportunities to test hypotheses about the role of CanSINEs in somatic functional diversity. Patterns of insertion also support species designations, affirming CanSINEs as systematic markers and confirming complex evolutionary processes including incomplete lineage sorting following rapid species divergence, hybridization and SINE mediated genome rearrangement.

## Methods

CanSINE distribution was assessed in one or more individuals representing each of the extant Felidae species including four subspecies of the domestic cat complex, *F. silvestris.* We also examined representative samples from five additional Feliformia families, Prionodontidae, Hyaenidae, Herpestidae, Eupleridae and Vivveridae. Taxa are listed in Additional file 1: Table S1. Commercial genomic DNA from *F. catus* was purchased from EMBD Biosciences Product No: 69235. Genomic DNA for the remaining taxa was extracted from blood and/or tissue samples using the Qiagen DNeasy Blood & Tissue Kit. All tissue samples for the Laboratory of Genomic Diversity were collected in full compliance with specific Federal Fish and Wildlife permits from the Conservation of International Trade in Endangered Species of Wild flora and Fauna: Endangered and Threatened Species, Captive Bred issued to the National Cancer Institute (NCI)-National Institutes of Health (NIH) (S.J.O. principal officer) by the U.S. Fish and Wildlife Services of the Department of the Interior.

### Genome mining

From a list of 322 felid SINEs identified during the initial *F. catus* whole genome annotation [8], select loci were retrieved from the March 2006 genome assembly on the UCSC genome browser (http://genome.ucsc.edu) and matched to corresponding cat chromosome locations using a *F. catus* genome browser, GARField (http://formerly at http://lgd.abcc.ncifcrf.gov/cgi-bin/gbrowse/cat/) [91]. Within the context of this study, each region is named for the UCSC genome browser scaffold from which the reference sequence was obtained (see Additional file 2: Table S2). Sixty regions containing feliform CanSINEs found in the *F. catus* whole genome sequence with homologous flanking sequence in *C. familiaris* were selected for amplification in all extant felids and five feliform outgroup taxa. Forward and reverse PCR primers were designed within 300 bp of the putative SINE insertion sites.

### Direct PCR, sequencing and cloning

Approximately 20 ng of extracted genomic DNA was used in each PCR reaction. All reactions consist of 0.1U of AmpliTaq DNA polymerase, 0.75 μM forward and reverse primer, 2.5 mM MgCl_2_, 0.2 mM of each deoxynucleotide triphosphate and the appropriate amount of 10X AmpliTaq Buffer II and water for a 20 μl reaction. Touchdown PCR conditions were 5 min at 94°C, 10 cycles of 30 sec at 94°C, 30 sec at 63°C* and 60 sec at 72°C, with a decrease in the annealing temperature at a rate of 0.5°C per cycle, followed by 30 cycles of 30 sec at 95°C, 30 sec at 58°C** and 60 sec at 72°C, then a final elongation step of 7 min at 72°C. **Final annealing temperatures varied from 50-64°C depending on the primer set. *Initial annealing temperatures were set to 5°C warmer than the final annealing temperature. To confirm amplification and assess the sizes of DNA fragments, 5 μl of PCR product was fractionated by gel electrophoresis in a 1.0% agarose gel containing ethidium bromide. Prior to cloning or sequencing, 20 μl of PCR product was purified using the ExoSAP protocol with 0.72 μl shrimp alkaline phosphatase (SAP) and 1.44 μl exonuclease I (ExoI) (Amersham Pharmacia, Piscataway, NJ).

Cycle sequencing reactions consisted of 0.25U BigDye® Terminator v3.1 Ready Reaction Mix, 0.075 μM primer, 5 μl of sequencing buffer (Applied Biosystems), 1.5 μl of purified PCR product and enough water for a 10 μl reaction. Cycle sequencing was performed under the following conditions: 94°C for 10 sec, 52°C for 5 sec, and 72°C for 2 min for 45 cycles. Products from cycle sequencing reactions were run on an ABI 3730 DNA Analyzer. Sequence results were visualized and edited with Sequencher v4.8 (GeneCodes).

Multiple gel electrophoresis bands or illegible preliminary sequencing traces were resolved by cloning PCR amplification products with the TOPO TA Cloning Kit (Invitrogen) followed by purification with the Qiagen GeneClean Kit according to manufacturer’s instructions. Cycle sequencing of the purified fragments was performed using 0.25U BigDye® Terminator v3.1 Ready Reaction Mix, 1 μl of forward or reverse M13 primer provided in the TOPO TA Cloning Kit, 5 μl of sequencing buffer (Applied Biosystems), 2.5 μl of purified PCR product and enough water for a 10 μl reaction. Cycle sequencing was performed under the following conditions: 94°C for 10 sec, 52°C for 5 sec, and 72°C for 4 min for 45 cycles.

### Scanning via SINE-to-SINE PCR and Cloning

A second SINE discovery method was adapted from a SINE-to-SINE amplification protocol [67] to allow identification of novel SINE loci in exotic Felidae species. Similar methods have been applied to illuminate human *Alu* loci [92,93]. Primers were developed that anneal to diagnostic motifs within the tRNA-related region of feliform CanSINEs: primer 1 (ATCAGACTCTTGATTTCAGCTCA), primer 2 (AGCTCAGGTCATGATCCCAGG), primer 3 (TCCGACTTCAGCCAGGTC), primer 4 (TGATGGCTCGGAGCCT) and primer 5 (TCCGACTTCGGCTCAGGTC). Single primer PCR was performed on approximately 20 ng of extracted genomic DNA from eight species representing the major Felidae lineages: *Neofelis nebulosa*, *Panthera onca*, *Pardofelis marmorata*, *Pardofelis badia, Leopardus guigna, Leopardus rufus, Octocolobus manul* and *Prionailurus viverrinus.* Reactions consisted of 0.1U of AmpliTaq DNA polymerase, 1.5 μM primer, 2.5 mM MgCl_2_, 0.2 mM of each deoxynucleotide triphosphate and the appropriate amount of 10X AmpliTaq Buffer II and water in a 20 μl total volume. PCR conditions were 5 min at 94°C, 40 cycles of 30 sec at 94°C, 30 sec at 54°C and 90 sec at 72°C followed by a final elongation step of 5 min at 72°C.

SINE-to-SINE amplifications resulted in a collection of DNA fragments flanked by head-to-head oriented CanSINE segments. To confirm amplification and assess the size range of DNA fragments, 5 μl of PCR product was fractionated by gel electrophoresis in a 1.0% agarose gel containing ethidium bromide. Prior to cloning, 15 μl of PCR product was purified using the ExoSAP protocol with 0.72 μl shrimp alkaline phosphatase (SAP) and 1.44 μl exonuclease I (ExoI) (Amersham Pharmacia, Piscataway, NJ). Isolation of SINE flanked fragments was completed using the TOPO TA Cloning kit (Invitrogen). Twelve to 24 clones from each query species were purified and sequenced following the protocol described in the previous section.

### Identification of novel informative SINE loci

Sequenced DNA fragments consisted of genomic sequence from the host species flanked at either end by the tRNA-related region of a feliform specific CanSINE insertion. After masking for low complexity repeats using RepeatMasker [70], the segments were aligned to the December 2008 10X *F. catus* whole genome sequence with the BLAST algorithm. When possible, the resulting homologous *F. catus* regions were extended 200 bp on either end, imported into Sequencher and aligned in appropriate contigs. Two screening strategies were then employed depending on insertion presence or absence status in *F. catus*. If SINEs identified in exotic species were absent in *F. catus*, primers were built around the putative insertion sites and all Felidae species were then amplified by direct PCR. Alternatively, if a SINE was initially identified a non-panthera lineage species and *F. catus*, primers were built around the putative insertion site and direct PCR was performed on a Pantherinae species. If the insertion is present in Pantherinae, then the insertion must have occurred in the ancestor of all Felidae. However, if the insertion is absent from Pantherinae, the insertion event must have occurred during the subsequent Felidae radiation. The site was then assessed by direct PCR and sequencing in all Felidae species as described in the previous section. After confirmation of amplification by gel electrophoresis, PCR products were purified and sequenced.

### Determining SINE presence or absence

A specific SINE insertion site is delimited by the exact sequence of the 6–20 base pair target site duplication (TSD). If a SINE is present the amplification product will include; the forward primer sequence, 5’ genomic sequence, one copy of the TSD, the SINE element, the second copy (duplicate) of the TSD, 3’ genomic sequence and the reverse primer sequence. If a SINE is absent, the amplification product will include; the primer sequences plus 5’ and 3’ genomic sequence bracketing one copy of the TSD sequence (canonical genomic DNA). Note that the absence of any PCR product signifies amplification failure and does not imply that the SINE is absent from the homologous region. Thus, criteria for successful amplification loci are 1) PCR products from *F. catus* include the target SINE insertion and therefore are about 200–400 base pairs larger than the amplification products of *C. familiaris* that lack the target SINE insertion, 2) the sequence of the TSD can be determined by examining sequence traces of *F. catus* 3) PCR products yielded sufficiently legible sequences such that SINE presence or absence at the TSD can be ascertained in at least 80% of the sample taxa.

### Evolutionary analysis of SINE subfamilies

Representing 87 full-length SINE insertion loci, 5’ tRNA regions were aligned using the MAFFT algorithm implemented in the Geneious software package version 5 [94-96]. Phylogenetic analyses were performed using minimum evolution, maximum parsimony and maximum likelihood methods. The Tamura-Nei plus gamma (TrN + G) model was selected as the optimal nucleotide substitution model for likelihood analyses using Modeltest with the AIC criterion [97,98]. Minimum evolution was implemented in Geneious [96] using the neighbor-joining algorithm, maximum parsimony was implemented using PAUP [99] and maximum likelihood was implemented in GARLI through the Lattice Project Grid computing system using the general time reversible model (nearest option to TrN) and a gamma distribution to account for among-site rate variation [100,101]. Bootstrap support values for all three analyses were obtained from 1000 repetitions. Genetic distances were obtained from the distance matrix calculated for the minimum evolution phylogeny. The mean rate of substitution for the tRNA-derived regions from each SINE subfamily and subtype as well as for all SINEs examined here were calculated by averaging the quotients: D/T where D is the genetic distance between each SINE pair and T minimum age of the most recent common ancestor of the lineages in which the pair of SINEs occur [60,66]. Tests for significance between substitution rates were calculated using the unpaired T-test, with significance at p < 0.05.

### Availability of supporting data

DNA sequences are catalogued in GenBank. Accession numbers are indicated in Additional tables 3 and 4. *Note, sequences under 200 base pairs cannot be catalogued but are available from the corresponding author. The phylogenetic data set supporting the results of this article is available in the TreeBase repository, at http://purl.org/phylo/treebase/phylows/study/TB2:S15822[102].

## Competing interests

The authors declare that they have no competing interests.

## Authors’ contributions

KWC conceived of the study, carried out the laboratory molecular genetic work, participated in the phylogenetic/statistical analyses and drafted the manuscript. DJ participated in the design and coordination of the study and critically revised the manuscript. WJ participated in the phylogenetic analyses and critically revised the manuscript. SO participated in the design of the study, critically revised the manuscript and provided final approval for publication. JPS conceived of the study, participated in its design and coordination, participated in the phylogenetic/statistical analyses and critically revised the manuscript. All authors read and approved the final manuscript.

## Supplementary Material

Additional file 1: Table S1 List of species used in study. Click here for file

Additional file 2: Table S2 PCR Primers used to amplify CanSINE containing regions. A) PCR primers flanking 29 feliform specific CanSINE insertions identified initial *F. catus* genome annotation (Pontius et al. [92]). B) PCR primers for 30 genomic loci containing informative CanSINE loci. Each primer pair is designated by the corresponding UCSC genome browser scaffold number (if known) and chromosome coordinates (if known). Click here for file

Additional file 3: Table S3 Genomic regions containing single CanSINE insertion events among feliforms. Target site duplications, distribution among taxa and corresponding GenBank accession numbers are indicated. Click here for file

Additional file 4: Table S4 Genomic regions containing multiple CanSINE insertion events among feliforms. Target site duplications, distribution among taxa and corresponding GenBank accession numbers are indicated. Click here for file

Additional file 5: Figure S1 Four unique CanSINE insertion events at locus 133135. Alignment of four CanSINE insertion events occurring at locus 133135 in the caracal (*Profelis caracal*), marbled cat (*Pardofelis marmorata*), ocelot lineage (genus: *Leopardus*) and bobcat (*Lynx rufus*). The homologous region without CanSINE from *F. catus* sequence in included as a reference. The *L. rufus* CanSINE is in reverse orientation, RC (reverse complement). Yellow-highlighted regions mark target site duplications and gray-shaded regions denote the A and B RNA polymerase III recognition sequences. Click here for file

Additional file 6: Figure S2 Six unique insertion events occurring at locus 212075. Alignment of six insertion events occurring at locus 212075 in the asian leopard cat lineage species of rusty spotted cat (*P. rubiginosus*), flat-headed cat (*P. planiceps*), Asian leopard cat (*P. bengalensis*); black-footed cat (*F. nigripes*); along with synapomorphies of the African golden cat clade (*P. caracal/aurata*), and the bay cat lineage. The homologous region without CanSINE from *F. catus* sequence in included as a reference. The insertions in the Asian leopard cat (*P. bengalensis*) and flat-headed cat (*P. planiceps*) are unfixed. Yellow-highlighted regions mark target site duplications and gray-shaded regions denote the A and B RNA polymerase III recognition sequences. Click here for file

Additional file 7: Figure S3 Alignment of Lynx lineage individuals at locus 106256. Alignment of Lynx lineage individuals at locus 106256 reveals a conserved Can-SINE in all species, a Can-SINE immediately adjacent in *L. pardinus* and some *L. lynx* individuals, and a different Can-SINE insertion that begins 260 bp 3’ of the conserved SINE in all *L. canadensis* and some *L. lynx* individuals. Each insertion has unique TSDs, as well as distinct SNPs in the tRNA related region. Yellow-highlighted regions mark target site duplications and gray-shaded regions denote the A and B RNA polymerase III recognition sequences. Click here for file

Additional file 8: Table S5 Distribution of locus 106265 CanSINEs among *Lynx pardinus*, *L. lynx*, and *L. canadensis* individuals. Ningxia, Qinghai, Yunnan are located in China. Click here for file

Additional file 9: Figure S4 Alignment of Lynx lineage individuals at locus 134463. Alignment of Lynx lineage individuals at locus 134463 reveals, a Can-SINE insert in *L. canadensis* (N = 7) and *L. lynx* (N = 3), yet absent from all 5 *L. pardinus* (N = 5). Yellow-highlighted regions mark target site duplications and gray-shaded regions denote the A and B RNA polymerase III recognition sequences. Click here for file

Additional file 10: Figure S5 Alignment of ocelot (Leopardus) lineage individuals at locus 133135. Alignment of ocelot (Leopardus) lineage individuals at locus 133135 reveals a Can-SINE insert in *L. pardalis*(N = 9) *L. tigrina* (N = 8), *O. geoffroyi* (N = 8) and *O. guigna* (N = 1) and *L.jacobita* (N = 1) individuals, yet absent from *L. colocolo* (N = 3) and *L. wiedii* (N = 2). Yellow-highlighted regions mark target site duplications and gray-shaded regions denote the A and B RNA polymerase III recognition sequences. Click here for file

Additional file 11: Figure S6 Alignment of DNA sequences from locus 161275. Alignment of DNA sequences from locus 161275 SINE amongst Asian leopard cat lineage species, with NADH5 haplotypes for each individual indicated in parentheses. Yellow-highlighted regions mark target site duplications and gray-shaded regions denote the A and B RNA polymersase III recognition sequences. The 2 rusty-spotted cat (*P. rubiginosa*) individuals have SINE sequences that differ from each other and are found amongst the asian leopard cat (*P. bengalensis*). Thus it cannot be determined whether the rusty-spotted cats acquired their SINEs through incomplete lineage sorting of ancestral polymorphisms or through hybridization with the Asian Leopard cat. Click here for file

Additional file 12: Figure S7 Alignment of a CanSINE insertion at locus 154966. Proliferation of the CanSINE at locus 154966 occurred during the initial Felidae radiation. Sequence from one species represents each lineage. The insertion is present in the domestic cat, asian leopard cat and lynx lineages. Yellow-highlighted regions mark target site duplications and gray-shaded regions denote the A and B RNA polymerase III recognition sequences. Click here for file

Additional file 13: Figure S8 Alignment of a CanSINE insertion at locus 214534. Proliferation of the CanSINE at locus 214534 occurred during the initial Felidae radiation. Sequence from one species represents each lineage. The insertion is present in all lineages except the Panthera lineage. Within the caracal lineage, the insertion is unfixed within the african golden cat species but absent in multiple caracal and serval individuals. Yellow-highlighted regions mark target site duplications and gray-shaded regions denote the A and B RNA polymerase III recognition sequences. Click here for file
