# Supplementary material for: The dynamic proliferation of CanSINEs mirrors the complex evolution of Feliforms
Source: BMC Evol Biol. 2014 Jun 20;14:137. doi: 10.1186/1471-2148-14-137 (PMC4084570; doi:10.1186/1471-2148-14-137)
Supplement: Additional file 1: Table S1 — List of species used in study. [file 1471-2148-14-137-S1.docx]

**Additional Table 1** List of species used in study

| Species | Common Name | Code | Lineage | Family |
| --- | --- | --- | --- | --- |
| Felis silvestris bieti | Chinese Desert Cat | FBI | Domestic Cat | Felidae |
| *Felis silvestris catus* | Domestic Cat | FCA | Domestic Cat | Felidae |
| *Felis chaus* | Jungle Cat | FCH | Domestic Cat | Felidae |
| *Felis silvestris libyca* | African Wild Cat | FLI | Domestic Cat | Felidae |
| *Felis margarita* | Desert Cat | FMA | Domestic Cat | Felidae |
| *Felis nigripes* | Black-footed Cat | FNI | Domestic Cat | Felidae |
| *Felis silvestris silvestris* | European Wild Cat | FSI | Domestic Cat | Felidae |
| *Leopardus colocolo* | Pampas Cat | LCO | Ocelot | Felidae |
| *Leopardus geoffroyi* | Geoffroy's Cat | OGE | Ocelot | Felidae |
| *Leopardus guigna* | Kodkod | OGU | Ocelot | Felidae |
| *Leopardus jacobita* | Andean Mt. Cat | OJA | Ocelot | Felidae |
| *Leopardus pardalis* | Ocelot | LPA | Ocelot | Felidae |
| *Leopardus tigrinus* | Tigrina | LTI | Ocelot | Felidae |
| *Leopardus wiedii* | Margay | LWI | Ocelot | Felidae |
| *Lynx canadensis* | Canada Lynx | LCA | Lynx | Felidae |
| *Lynx lynx* | Eurasian Lynx | LLY | Lynx | Felidae |
| *Lynx pardinus* | Iberian Lynx | LYP | Lynx | Felidae |
| *Lynx rufus* | Bobcat | LRU | Lynx | Felidae |
| *Panthera leo* | Lion | PLE | Panthera | Felidae |
| *Panthera onca* | Jaguar | PON | Panthera | Felidae |
| *Panthera pardus* | Leopard | PPA | Panthera | Felidae |
| *Panthera tigris* | Tiger | PTI | Panthera | Felidae |
| *Panthera uncia* | Snow Leopard | PUN | Panthera | Felidae |
| *Neofelis nebulosa* | Clouded Leopard | NNE | Panthera | Felidae |
| *Pardofelis badia* | Bay Cat | PBA | Bay Cat | Felidae |
| *Pardofelis marmorata* | Marbled Cat | PMA | Bay Cat | Felidae |
| *Pardofelis temminckii* | Asian Golden Cat | PTE | Bay Cat | Felidae |
| *Otocolobus manul* | Pallas Cat | OMA | Leopard Cat | Felidae |
| *Prionailurus bengalensis* | Asian Leopard Cat | PBE | Leopard Cat | Felidae |
| *Prionailurus planiceps* | Flat-headed Cat | IPL | Leopard Cat | Felidae |
| *Prionailurus rubiginosus* | Rusty Spotted Cat | PRU | Leopard Cat | Felidae |
| *Prionailurus viverrinus* | Fishing Cat | PVI | Leopard Cat | Felidae |
| *Profelis aurata* | African Golden Cat | PAU | Caracal | Felidae |
| *Profelis caracal* | Caracal | CCA | Caracal | Felidae |
| *Profelis serval* | Serval | LSE | Caracal | Felidae |
| *Puma concolor* | Puma | PCO | Puma | Felidae |
| *Puma yagouaroundi* | Jaguarondi | HYA | Puma | Felidae |
| *Acinonyx jubatus* | Cheetah | AJU | Puma | Felidae |
| *Helogale hirtula* | Dwarf mongoose | HPA | Other Feliformia | Herpestidae |
| *Genetta genetta* | Genet | GGE | Other Feliformia | Viverridae |
| *Hyaena hyaena* | Hyena | HHY | Other Feliformia | Hyaenidae |
| *Prionodon linsang* | Banded Linsang | PLI | Other Feliformia | Prionodontidae |
| *Cryptoprocta ferox* | Fossa | CFE | Other Feliformia | Eupleridae |
