# Supplementary material for: The dynamic proliferation of CanSINEs mirrors the complex evolution of Feliforms
Source: BMC Evol Biol. 2014 Jun 20;14:137. doi: 10.1186/1471-2148-14-137 (PMC4084570; doi:10.1186/1471-2148-14-137)
Supplement: Additional file 2: Table S2 — PCR Primers used to amplify CanSINE containing regions. A) PCR primers flanking 29 feliform specific CanSINE insertions identified initial F. catus genome annotation (Pontius et al. [92]). B) PCR primers for 30 genomic loci containing informative CanSINE loci. Each primer pair is designated by the corresponding UCSC genome browser scaffold number (if known) and chromosome coordinates (if known). [file 1471-2148-14-137-S2.docx]

**Table S2** A) PCR primers flanking 29 feliform specific CanSINE insertions identified initial *F. catus* genome annotation (Pontius et al 2007). B) PCR primers for 30 genomic loci containing informative CanSINE loci. Each primer pair is designated by the corresponding UCSC genome browser scaffold number (if known) and chromosome coordinates (if known).

| Scaffold | Forward Primer 5’-3’ | Reverse Primer 5’-3’ | Chromosome Position |
| --- | --- | --- | --- |
| 4743  95892 | agtcaggacacctggtttgg  ctgtaccattttctcttttcat | GTGATGTTGGTAATTATATCCTCCC  TCATATATCTGACAAAGGATTA | C1: 124821471- 124931865  X: 106422338 - 106431094 |
| 99463 | gtttattccttctggatttcttcag | GAAAGTTCACAGAAAAGCTAGATGA | B4: 88534201 - 88540012 |
| 101187 | cttaacctctctgtgcc | ATCATTGAGGTAATCCCT | C2: 125094248 - 125106032 |
| 106265 | taggaagatattgttatggaaag | tgagtaatcaaccttggtatt | C1:47971606-47972269 |
| 115304 | tcatataacagtcccactctgg | agcatatgaatgtgcacatg | Un3: 10045041 - 10084868 |
| 117885 | acatggctgatggaaaact | AAGTTTGTTGTGTTCCTGAGA | Un36: 4121470 - 4126051 |
| 122236 | tcattggctaagaggttg | tctctgttgccagaccttc | Un9: 2561094 - 2563938 |
| 131713 | ttggaaatctcactgtcttttctc | GTGTGCTTCCATAACAAGGC | D2: 18001674 - 18212792 |
| 134292 | tgtaatgagaaYataaaatttaactataat | GCAAAGCTTTTAACAATTTATTC | D1: 112776152 - 113004154 |
| 135256 | gggagtgactgctaatgagta | TTTGAGAATGCCCATTTC | D1: 135487868 - 135678524 |
| 136949 | cagaaaactccagattttctactt | atttaggtctttgatccatttt | D1: 23281897 - 23598106 |
| 136982 | aattctcatcttttctgaattgag | CCTCAAGCCATCCTACAGTAA | D1: 25280168 - 25429612 |
| 145617 | agatgctctcctcaccct | tcagcaaccttggtgaga | B3: 101408163 - 101878758 |
| 145754 | ctctgtacttttgctcaattt | tggccaagaaagctaatt | B3: 105227423 - 105339802 |
| 150913 | cttcagaacacatgcaagg | GCTGAAACCATTATCAGTTCA | E2: 66170238 - 66723524 |
| 151787 | GTGCCAAACCACAGCCT | ATCCTGAGTCCCAAGCTGTT | Un9: 1208555 - 1276996 |
| 158776 | gtaaaatcatccctaaacaaatt | atttaagatcagtggctcatt | A3: 75423320 - 75426336 |
| 164336 | actaaatttttaaatagctctaaa | TGCCCATTTTTTAACCTA | A3: 5375696 - 5567150 |
| 170176 | tctgccttttgttccatagactt | gctgggaagaaggtgagtct | C2: 62008241 - 62329430 |
| 174511 | caattttatgaggaaaataatatctctat | TTGGGAGGCTCATTTTCT | Un13: 9741721 - 9761046 |
| 179189 | ccagggctttaatgtaaagtttatt | GCTAAATGCAACCACTTGC | B1: 3729770 - 3958145 |
| 180515 | tagtatcatctggaaRaatgc | CTGTGGACACCTCTTATCTC | A1: 249714913 - 249755626 |
| 194731 | ctgagcagacctggattc | TTAGAACACTTYATGAAGGT | A2: 200196491 - 200445527 |
| 199572 | acatgcattcctctttgaat | TAATCAATACAGGTACCAGTGACTC | D4: 33412603 - 33662084 |
| 203536 | tttcccaaaactcctttcttgt | acatgtttccatggccca | X: 69879474 - 70012608 |
| 204133 | acctactcagtaacctaattctgc | GCTAATTAATTTCAAATATTTCCAT | X: 93071968 - 93626326 |
| 213652 | tgcatctgagagatcaaaaat | TCAGGGAGGGGATGTTG | E1: 57845691 - 58503085 |
| 217179 | ataggcattttattttgaattt | GAAAAGGAAATTCAAATGG | X: 65114741 - 65370428 |

**A.**

| Scaffold | Forward Primer 5'-3' | Reverse Primer 5'-3' | Chromosome Position |
| --- | --- | --- | --- |
| n/a | ACTACATGTCCCTACCATTCA | TAAATTACAAGATCATTTTGGATA | chrA1:248917944-248918545 |
| n/a | TTCCCTAACAGGTTGAAATG | TATGAAATTTATTGTCAAATTGGT | chrB1:32807332-32807799 |
| n/a | CAGGTGAAGCTACCTCCACTA | AGTCCCTGGCACTGTGC | chrC1:181748497-181748747 |
| n\a | TGGTTTATGCAGAGAACTGGTA | CATCACGCAGCCACAAA | chrD4:80722469-80722612 |
| 782 | GTGCTTAGCTCTGAGTGTTGGA | GAGACTGGGAGCCTCCAGA | chrF1:82759656-82759439 |
| 5313 | TGAAGGTGGCAGTGCATGT | ACCTGTGAATCTAGGCTCCCTA | chrA3:7066904-7067187 |
| 73133 | GCTGTACATATTATGGTAGTCAGTTC | CAAGTGGTCTCATGTATTGACA | chrA1:78064263-78064761 |
| 125972 | ATGGCTAAAAGTCTACTTCAGACT | CAGAGGCCACGTTAGACATTA | chrC1:74363856-74363642 |
| 130416 | AGCTTGTCATACATCAGACTTCA | TAGCGCATTTATCTCTGTGTTCT | chrB4:9912326-9912585 |
| 133135 | TAATGCAAGTGTGTCTCATTCTA | ACAGTAGGGTCCTGCTTCAG | chrD2:92520918-92520698 |
| 134463 | GATGTCTCTCAAATGATTCATGTCT | AGTGTCTTACGTTTCTGAATCTCTTCT | chrD1:118666712-118666980 |
| 139216 | CTGAACTTATCCGTCTGTGATCAATA | CCCAGATTGAGTCTCTTGAGATCT | chrB4:109463719-109463931 |
| 146417 | AACTAAACAGAAGATCCACAGG | TGATTGGAGAACATGCTTG | unknown |
| 150853 | AGGCTTCAAAGTCTGGTGT | TCTTCTGTCACAGAGTTGACC | chrE2:65441081-65441305 |
| 150890 | CATGGACAAACTAGGAATGCC | GCTCCAACACCCCACTGT | chrA2:162899126-162899564 |
| 150951 | CCTTGACTGGCCAGTTGACT | AGTCACTCTGAGTCTTACTGAAATGA | chrE2:71800542-71800192 |
| 154966 | TAGATTTTCCTTCCAATTTCCTATGTAC | AGCTTGACACCGTCCTAATTCT | unknown |
| 161275 | ATTGCTGTAACAATCTACATTAAGAAAC | CCAAGAGGTTAGCAATTTCCAT | chrC1:184822652-184823028 |
| 168013 | ACTGCTGTATTCTTAGGACCCT | GTCCATCTTGTCTGGTGTGA | unknown |
| 188620 | ACTATTTAAGCTGGGTCTGGA | CTTTGTGATGCCTCTGAACA | chrB2:122398146-122398467 |
| 203464 | ATGCGCCYTGCATACTG | GCCCATAAACTGGATAATCAGTC | chrX:73848142-73848698 |
| 206537 | CTCATTAGAAACGTGATGTCTG | GTGCTACCCGGAGGAA | chrUn2:3605781-3605984 |
| 212075 | CCATGACCCTATCTTTAAATAAGAGC | GCAATGTGTTCCACATCTTAGAGTT | chrD2:7263045-7262816 |
| 212331 | AGACTCTGTGCAATAGCTCATTTA | GCATTCTCCGGCATTTATAT | unknown |
| 212733 | ACTACCTTGAGAAAGCAATATGCT | ACAAGGTATGACCATGCAAG | chrB3:91276896-91276402 |
| 213566 | ACGCTCATCACCCAACTGT | CAGGTCACAACGGAGGTCA | chrE2:66187777-66188092 |
| 213798 | TGCCAATCAAAGATGTAAGTTACA | AGTAAATGGTGTATTGACATATGGGA | chrD3:47912789-47913122 |
| 214534 | TGGCCAAATGCGAGAAGTA | TGTCATAAGCAAGTTTCCATGC | chrA3:41096151-41096453 |
| 215112 | TCAGATATACATAGCACTGCTATAAGAAT | AAGGATAGGCATCAGGTTAGG | chrC2:108111706-108111935 |
| 216162 | ATGACACAATTCAGGACTTACA | AAGTATAATACTCACCCATCTGAAG | chrUn12:15996523-15996731 |

**B.**
