# Supplementary material for: The dynamic proliferation of CanSINEs mirrors the complex evolution of Feliforms
Source: BMC Evol Biol. 2014 Jun 20;14:137. doi: 10.1186/1471-2148-14-137 (PMC4084570; doi:10.1186/1471-2148-14-137)
Supplement: Additional file 3: Table S3 — Genomic regions containing single CanSINE insertion events among feliforms. Target site duplications, distribution among taxa and corresponding GenBank accession numbers are indicated. [file 1471-2148-14-137-S3.docx]

**Table S3** Genomic regions containing single CanSINE insertion events among feliforms.

| UCSC Scaffold | Chromosome Location  Relative to *Felis catus* genome | Accession Numbers | Target Site Duplication Of 5’-3’ Loci | Taxonomic Distribution of Sites within Feliformia |
| --- | --- | --- | --- | --- |
| 4743 | C1: 124821471- 124931865 | KJ931671-KJ931704 | AAAGCAAAGTGTT | Feliformia |
| 95892 | X: 106422338 - 106431094 | KJ931705-KJ931741 | ACTCATTTATTT | Feliformia |
| 115304 | Un3: 10045041 - 10084868 | KJ932036-KJ932069 | TAAAAAGACATTTGTAG | Felidae/Prionodontidae |
| 117885 | Un36: 4121470 - 4126051 | KJ931742-KJ931771 | AAAAAACAATAAC | Feliformia |
| 135256 | D1: 135487868 - 135678524 | KJ932070-KJ932084 | AAAAGTATATAATT | Felidae |
| 136949 | D1: 23281897 - 23598106 | KJ932085-KJ932100 | GAAAAGATTATTCTT | Feliformia |
| 136982 | Unknown | KJ932101-KJ932135 | AAACCAGGACTCTGGGT | Feliformia |
| 145617 | B3: 101408163 - 101878758 | KJ931772-KJ931799 | AAGAGCAAAT | Feliformia |
| 145754 | B3: 106130389 - 106130764 | KJ931800-KJ931831 | GATCCTATATRCTRTTTA | Feliformia |
| 150913 | E2: 66170238 - 66723524 | KJ931832-KJ931859 | AAAAGGAGTCCTTCT | Feliformia |
| 151787 | Un9: 1208555 - 1276996 | KJ931860-KJ931891 | Undefined | Feliformia |
| 158776 | A3: 75423320 - 75426336 | KJ931892-KJ931917 | AAAATAAAA | Feliformia |
| 170176 | C2: 62008241 - 62329430 | KJ931918-KJ931943 | AAGGATTTCAATGTTA | Feliformia |
| 174511 | Unknown | KJ932136-KJ932171 | ATCAAATGAAAG | Feliformia |
| 179189 | B1: 3729770 - 3958145 | KJ931944-KJ931963 | AAATTCT | Feliformia |
| 180515 | A1: 249714913 - 249755626 | KJ932172-KJ932205 | GTAGAGTCTTGG | Feliformia |
| 194731 | A2: 200196491 - 200445527 | KJ931964-KJ931967 | AAGATTCACTCCACTTCA | Feliformia |
| 203536 | X: 69879474 - 70012608 | KJ931968-KJ932000 | AAAGGATTTCAATGTTA | Feliformia |
| 217179 | X: 65114741 - 65370428 | KJ932001-KJ932035 | AAGACTTTCATCGG | Feliformia |
| unmatched | D4:80722469-80722612 | KJ932206-KJ932207 | TAATGGCTCACA | *Leopardus guigna/geoffroyi* |
| 206537 | Un2:3605781-3605984 | KJ932208-KJ932232 | CGAGCCCCGCGTC | *Leopardus guigna/geoffroyi/tigrinus* |
| 213566 | E2:66187777-66188092 | KJ932233-KJ932254 | TGTTAAGAGGAGTCAATTGCC | *Otocolobus manul* |
| 212331 | Unknown | KJ932255-KJ932257 | TCAAAAGCAGTGAATCTA | *Otocolobus manul* |
| 215112 | C2:108111706-108111935 | KJ932258-KJ932269 | Undefined | *Otocolobus manul* |
| 213798 | D3:47912789-47913122 | KJ932270-KJ932304 | GCTAGCACA | *Leopardus guigna/geoffroyi/tigrinus* |
| 146417 | Unknown | KJ932305-KJ932336 | GAACACATGGAATA | *Pardofelis badia* |
| 216162 | Un12:15996523-15996731 | KJ932337-KJ932348 | TCAAAGATTATT | *Pardofelis marmorata* |
| 5313 | A3:7066904-7067187 | KJ932349-KJ932385 | GACATAAGCATGG | *Panthera onca* |
| 782 | F1:82759656-82759439 | KJ932386-KJ932410 | TTCCCTGTGGGT | *Panthera* genus |
| unmatched | B1:32807332-32807799 | KJ932411 | AGAAAATGAACAAACA | *Panthera onca* |
| 161275 | C1:184822652-184823028 | KJ932412-KJ932460 | TTAAAAATTGTTTTGA | *Prionailurus rubiginosus/bengalensis/viverrinus* |
| 125972 | C1:74363856-74363642 | KJ932461-KJ932477 | AAAGAGCTAGCTTG | *Prionailurus viverrinus* |
| 168013 | Unknown | KJ932478-KJ932504 | AAAAGACATAC | *Prionailurus viverrinus* |
| unmatched | C1:181748497-181748747 | KJ932505-KJ932535 | CTCGTACTYTTATC | *Prionailurus planiceps/bengalensis*  */viverrinus* |
| 130416 | B4:9912326-9912585 | KJ932536-KJ932559 | AGGATCAAAA | *Prionailurus genus* |
| 188620 | B2:122398146-122398467 | KJ932560-KJ932590 | GTAAGGGGTGGT | *Panthera onca* |
| 214534 | chrA3:41096151-41096453 | KJ932591-KJ932613 | GCAAAAAATA | *All Felidae except Panthera and Caracal Lineages* |
| 154966 | Unknown | KJ932614-KJ932630 | AGAAAATGTGGTAGA | *Lynx, Asian Leopard Cat and Domestic Cat Lineages* |
