# Supplementary material for: The dynamic proliferation of CanSINEs mirrors the complex evolution of Feliforms
Source: BMC Evol Biol. 2014 Jun 20;14:137. doi: 10.1186/1471-2148-14-137 (PMC4084570; doi:10.1186/1471-2148-14-137)
Supplement: Additional file 4: Table S4 — Genomic regions containing multiple CanSINE insertion events among feliforms. Target site duplications, distribution among taxa and corresponding GenBank accession numbers are indicated. [file 1471-2148-14-137-S4.docx]

**Table S4** Regions containing multiple CanSINE insertion events among feliforms.

| UCSC Scaffold | Chromosome Location  Relative to *Felis catus* genome | Accession Numbers | Target Site Duplication  Of 5’-3’ Loci | Taxonomic Distribution of Sites within Feliformia |
| --- | --- | --- | --- | --- |
| 99463 | B4: 88534201 - 88540012 | KJ932631- KJ932663 | AAAACAGGRTTAA | Feliformia |
|  |  |  | TTAAAACA | Felis nigripes |
| 101187 | C2: 125094248 - 125106032 | KJ932664- KJ932697 | TCCCATAA | Feliformia |
|  |  |  | TTTAAAT | Caracal serval |
|  |  |  | TTAAAAAGAGA | Puma yagouaroundi |
| 106265 | B1: 191469873 - 191484794 | KJ932698- KJ932727 | AAAAGTAAAAGAGATTA | Feliformia |
|  |  |  | AAAAAAAAATACA | Lynx canadensis/lynx |
|  |  |  | AAAATAAAAATTA | Lynx lynx/pardinus |
|  |  |  | atgagattat | Pardofelis marmorata |
| 122236 | Un9: 2561094 - 2563938 | KJ932728- KJ932752 | AAAAACTGAATTCAT | Felidae |
|  |  |  | TTTAAAAACTGAATTCAT | Leopardus colocolo |
| 131713 | D2: 18001674 - 18212792 | KJ932753- KJ932786 | AGAAATGGTGT | Feliformia |
|  |  |  | Undefined | Cryptoprocta ferox |
| 134292 | D1: 112776152 - 113004154 | KJ932787- KJ932818 | TTTTTGAACA | Feliformia |
|  |  |  | AAAACGTTGT | Panthera tigris/uncia |
| 164336 | A3: 5375696 - 5567150 | KJ932819- KJ932853 | AAAGCAGTGGAACTA | Feliformia |
|  |  |  | aaataatttta | Prionailurus planiceps |
| 199572 | D4: 33412603 - 33662084 | KJ932854- KJ932881 | AGACATTTATATCTACCA | Feliformia |
|  |  |  | aaagattccta | Hyaena hyaena |
|  |  |  | agatactctggtag | Neofelis |
| 213652 | E1: 57845691 - 58503085 | KJ932882- KJ932914 | AAAATGYAGCC | Feliformia |
|  |  |  | GAGTTTA | *Puma yagouaroundi* |
| 204133 | X: 93071968 - 93626326 | KJ932915- KJ932947 | AAGACTTCCATTGGA | Feliformia |
|  |  |  | GAGTTCAAG | Lynx lynx |
| 133135 | D2:92520918-92520698 | KJ932948- KJ932985 | AAAATAAAGATTTATTT | *Leopardus jacobita/pardalis*  */tigrinus/geoffroyi/guigna* |
|  |  |  | TAAAG | *Lynx rufus* |
|  |  |  | AAAATAAAGATTT | *Profelis caracal* |
|  |  |  | TTTAAAATAAAGATT | *Pardofelis marmorata* |
| 212075 | D2:7263045-7262816 | KJ932986- KJ933003 | TATAAGGGAGG | Bay Cat Lineage |
|  |  |  | Undefined | *Profelis caracal/aurata* |
|  |  |  | AAGAAGTAAAGCTTTA | *Felis nigripes* |
|  |  |  | AACTTGGT | *Prionailurus rubiginosus* |
|  |  |  | AAGGTATAAGGG | *Prionailurus planiceps* |
|  |  |  | AAGAAGTAAGGCTT | *Prionailurus bengalensis* |
| 150951 | E2:71800542-71800192 | KJ933004- KJ933041 | AAAAAGCCATTCTA | Asian Leopard Cat Lineage |
|  |  |  | TTAAAAATAATGTTCCC | *Felis nigripes* |
|  |  |  | TAAGACCCAAAGATTAT | Ocelot Lineage |
| Unmatched | A1:248917944-248918545 | KJ933042- KJ933077 | GTTTTAAATAATTT | Panthera Lineage |
|  |  |  | AAAGTAACA | *Pardofelis marmorata* |
|  |  |  | AAAGATACATGTTCA | *Prionailurus genus* |
| 105890 | A2:162899126-162899564 | KJ933078- KJ933109 | TAAACTTA | *Otocolobus manul* |
|  |  |  | TCATGATG | Caracal Lineage |
|  |  |  | TTAGCC | Domestic Cat Lineage |
| 203464 | X:73848142-73848698 | KJ933110- KJ933140 | TAAGAATCT | *Felis silvestris/margarita* |
|  |  |  | GAATCTATTG | *Felidae* |
| 139216 | B4:109463719-109463931 | KJ933141- KJ933156 | AATAAAATAGGCAATATCA | Lynx Lineage |
|  |  |  | TGGTGAGGATATTTTGA | *Otocolobus manul* |
| 212733 | B3:91276896-91276402 | KJ933157- KJ933189 | TTTCATCAGATTTT | *Otocolobus manul* |
|  |  |  | AAAAGCCTT | *Neofelis* |
| 73133 | A1:78064263-78064761 | KJ933190- KJ933221 | TATATAGACTTTTT | *Otocolobus manul* |
|  |  |  | AATCACATCCA | *Lynx canadensis* |
| 150853 | E2:65441081-65441305 | KJ933222- KJ933254- | AAAATGGAAGTTGCTA | *Prionailurus* genus |
|  |  |  | TACACACCA | *Pardofelis marmorata* |
| 134463 | D1:118666712-118666980 | KJ933255- KJ933284 | AAATTTAA | *Prionailurus genus* |
|  |  |  | AGTCAAGAAG | *Lynx canadensis/lynx* |
