# Supplementary material for: The dynamic proliferation of CanSINEs mirrors the complex evolution of Feliforms
Source: BMC Evol Biol. 2014 Jun 20;14:137. doi: 10.1186/1471-2148-14-137 (PMC4084570; doi:10.1186/1471-2148-14-137)
Supplement: Additional file 6: Figure S2 — Six unique insertion events occurring at locus 212075. Alignment of six insertion events occurring at locus 212075 in the asian leopard cat lineage species of rusty spotted cat (P. rubiginosus), flat-headed cat (P. planiceps), Asian leopard cat (P. bengalensis); black-footed cat (F. nigripes); along with synapomorphies of the African golden cat clade (P. caracal/aurata), and the bay cat lineage. The homologous region without CanSINE from F. catus sequence in included as a reference. The insertions in the Asian leopard cat (P. bengalensis) and flat-headed cat (P. planiceps) are unfixed. Yellow-highlighted regions mark target site duplications and gray-shaded regions denote the A and B RNA polymerase III recognition sequences. [file 1471-2148-14-137-S6.pdf]

```
                           10        20        30        40        50        60        70        80        90       100       110       120
                                  5′ TSDs              Pol A Box                                          Pol B Box
Consensus            GATGTATTAAGAAGTAAACCTT-------------------------------------------------------------------------------------------------------------
F. catus             ......................-------------------------------------------------------------------------------------------------------------
P. planiceps 1       ......................-------------------------------------------------------------------------------------------------------------
P. planiceps 2       ......................-------------------------------------------------------------------------------------------------------------
P. bengalensis 1     ......................-------------------------------------------------------------------------------------------------------------
P. bengalensis 2     ......................--GGGGCGC-TGGGTGGCGCAGTCGGTTAAGCATCCGACTTCAGCCAGGTCACGATCTCGCGGTCCGTGAGTTCGAACCCCGCGTCGGGCTCTGGGCTGATGGCTCAGA
P. rubiginosus       ......................-------------------------------------------------------------------------------------------------------------
P.caracal/aurata     ......................-------------------------------------------------------------------------------------------------------------
Bay Cat Lineage      ......................-------------------------------------------------------------------------------------------------------------
F. nigripes          ......................TAGGGGCGCCTGGGTGGCGCAGTCGGTTAAGCGTCCGACTTCAGCCAGGTCACGATCTCGCGGTCCGTGAGTTCGAGCCCCGCGTCGGGCTCTGGGCTGATGGCTCAGA

                          140        150       160       170       180       190       200       210       220       230      240       250
                                          >>>>>(CT) rich region>>>>>>>>>>>>>>>>>>>>>>>>>>>>>>>>>>***********Poly-A/T Tail*****************************
Consensus            -----------------------------------------------------------------------------------------------------------------------------------
F. catus             -----------------------------------------------------------------------------------------------------------------------------------
P. planiceps 1       -----------------------------------------------------------------------------------------------------------------------------------
P. planiceps 2       -----------------------------------------------------------------------------------------------------------------------------------
P. bengalensis 1     -----------------------------------------------------------------------------------------------------------------------------------
P. bengalensis 2     GCCTGGAGCCCGTTTCCGATTCTGTGTCTCCCTCTCTCTCTGCCCTCCCCCGTTCATGCCCTGTCTCTCTCTGTCCCAAAAATAAAATAAAACGTGAAAAAAAAA-TT-----------------C-----
P. rubiginosus       -----------------------------------------------------------------------------------------------------------------------------------
P.caracal/aurata     -----------------------------------------------------------------------------------------------------------------------------------
Bay Cat Lineage      -----------------------------------------------------------------------------------------------------------------------------------
F. nigripes          GCCTGGAGCCTGTTTCCGATTCTGTGTCTCCCTCTCTCTCTGCCCTCCCCCGTTCATGCTCTGTCTCTCTCTGTCCCAAAAAT-AAATAAATGTTGAAAAAAAAAATTTTTTTAAACAAAAAAAATCAAAAA

                          270        280       290       300       310       320       330       340       350       360       370       380
                     ***********   3′ TSDs                                      5′ TSD          Pol A Box
Consensus            ----------------------------TAGCACGATGGGACAGAAGTTTATTTAAAAAGCG---------------------------------------------------------------------
F. catus             ----------------------------..................................---------------------------------------------------------------------
P. planiceps 1       ----------------------------..................................---------------------------------------------------------------------
P. planiceps 2       ----------------------------..................................---------------------------------------------------------------------
P. bengalensis 1     ----------------------------..................................---------------------------------------------------------------------
P. bengalensis 2     ------------TAAAGAAGTAAGG---..................................---------------------------------------------------------------------
P. rubiginosus       ----------------------------..................................AACTTGGTGGGGCGCCTGGGTGGCGCAGTCGGAGAAGCGTCCGACTTTGATGAGTTACCATCTTGCGGGC
P.caracal/aurata     ----------------------------..................................---------------------------------------------------------------------
Bay Cat Lineage      ------------------------------.........--.....................---------------------------------------------------------------------
F. nigripes          AAATAAAAAAAATAAAGAAGTAAAGCTT.....S..G.........................---------------------------------------------------------------------

                          400        410       420       430       440       450       460       470        480       490       500       510
                         Pol B Box                                                 >>>>>>>>>>>>>>>>                                   5′ TSDs
Consensus            ---------------------------------------------------------------------------------GTAATGGTAAAGGTGATTTCTCACTAAAAGAGGTATAAGGGA-------
F. catus             -------------------------------------------------------------------------------AT..........................................-------
P. planiceps 1       ---------------------------------------------------------------------------------..........................................-------
P. planiceps 2       ---------------------------------------------------------------------------------.............................A............GGGGCGC
P. bengalensis 1     ---------------------------------------------------------------------------------..........................................-------
P. bengalensis 2     ---------------------------------------------------------------------------------..........................................-------
P. rubiginosus       TGTTAATGTGGAGCCCCTCGTCTGCCTCTGGACTGAAGGCTTAGACCCTGGAGCCTGTTTCCGACTCTGTGTCTCCCTCTC--........................................-------
P.caracal/aurata     ---------------------------------------------------------------------------------..........................................GGGGCCC
Bay Cat Lineage      ---------------------------------------------------------------------------------.........................................GGGGGGC
F. nigripes          ---------------------------------------------------------------------------------..........................................-------
```

```
                                530       540       550       560       570       580       590       600       610       620       630       640
                           Pol A Box                                                                                 Pol B Box
Consensus             ---------------------------------------------------------------------------------------------------------------------------------
F. catus              ---------------------------------------------------------------------------------------------------------------------------------
P. planiceps 1        ---------------------------------------------------------------------------------------------------------------------------------
P. planiceps 2        CTGGGTGGCGCAGTCGGTTAAGCGTCCGACTTCAGCCAGGTCA--------------------------CGATCTCGCGGTCCGGGAGTTCGAGCCCCGCGTCGGGCTCTGGGCTGATGGCTCAGAGCC
P. bengalensis 1      ---------------------------------------------------------------------------------------------------------------------------------
P. bengalensis 2      ---------------------------------------------------------------------------------------------------------------------------------
P. rubiginosus        ---------------------------------------------------------------------------------------------------------------------------------
P.caracal/aurata      CTGGGTGGCGCAGTGGGTTAAGCGTCCGACTTCAGCCAGGTCACGATCTCGCAATCTCGCGGGATCTCGCGATCTCGCGGTCCGTGAGTTCGAGCCCCGCGTCAGGCTCTGGGCTGATGGCTCGGAGCC
Bay Cat Lineage       CTGGGTGGGGCAGTCGGTTAAGGGTCCGAATTCAGCCAGGTCA--------------------------CGATCTCGCGGTCCGGGAGTTTGAGCCCCGGCTCAGGCTTTGGGGTGATGGCTTAGCGCC
F. nigripes           ---------------------------------------------------------------------------------------------------------------------------------

                               660       680       690       700       710       720       730       740       750       760       770       780
                                                 >>>>>>>>>>>>>>>>>>>(CT) rich region>>>>>>>>>>>>>>>>>>  *****Poly-A/T Tail********* [3' TSDs ]
Consensus             ---------------------------------------------------------------------------------------------------------------------------AAATTTTGCAA
F. catus              ---------------------------------------------------------------------------------------------------------------------------...........
P. planiceps 1        ---------------------------------------------------------------------------------------------------------------------------...........
P. planiceps 2        TGGAGCCTGTTTCCGATTCTGTGTCTCCCTCTCTCTCTGCCCCTCCCCCGTTCATGCTCTGTCTCTCTCTGTCCCCAAAATAAATAAATAAACGT-GAA-----AGAGGTATAAGGG----...........
P. bengalensis 1      ---------------------------------------------------------------------------------------------------------------------------...........
P. bengalensis 2      ---------------------------------------------------------------------------------------------------------------------------...........
P. rubiginosus        ---------------------------------------------------------------------------------------------------------------------------...........
P.caracal/aurata      TGGAGCCTGTTTCCGATTCTGTGTCTCCCTCTCTCTCTGCCCCTCCCCCGTTCATGCTCTGTCTCTCTCTGTCCCAAAAAATAAATAAATAAACGTTGAAAAAAAAAAAAT------------...........
Bay Cat Lineage       TGGAGCCTGTTTCCGATTCTGTGTCTCCCTCTCTCTCTGCCCCTCCCCCGTTCATGGTCTGTCTCTCTCTCTCCCCCAAATAAATAAATAAATGTTAAAAAAAAAAATTATATAAGGGAGG-...........
F. nigripes           ---------------------------------------------------------------------------------------------------------------------------...........
```

Figure S2 Alignment of six unique insertion events occurring at locus 212075 in the rusty spotted cat (*P. rubiginosus*), flat-headed cat (*P. planiceps*), Asian Leopard cat species (*P. bengalensis*), the caracal and black-footed cat (*F. nigripes*) along with synapomorphies of the African golden cat clade (*P. caracal/aurata*), and the Bay Cat lineage. The insertions in the Asian Leopard cat species (*P. bengalensis*) and flat-headed cat (*P. planiceps*) are unfixed. Yellow-highlighted regions mark target site duplications and gray-shaded regions denote the A and B RNA polymerase III recognition sequences.
