# Supplementary material for: The dynamic proliferation of CanSINEs mirrors the complex evolution of Feliforms
Source: BMC Evol Biol. 2014 Jun 20;14:137. doi: 10.1186/1471-2148-14-137 (PMC4084570; doi:10.1186/1471-2148-14-137)
Supplement: Additional file 8: Table S5 — Distribution of locus 106265 CanSINEs among Lynx pardinus, L. lynx, and L. canadensis individuals. Ningxia, Qinghai, Yunnan are located in China. [file 1471-2148-14-137-S8.docx]

**Table S5.** Distribution of locus 106265 CanSINEs among *Lynx pardinus*, *L. lynx*, and *L. canadensis* individuals. Ningxia, Qinghai, Yunnan are located in China.

| **Sample-ID** | **Origin** | **SINE** | **Sample-ID** | **Origin** | **SINE** |  |
| --- | --- | --- | --- | --- | --- | --- |
|  |  |  |  |  |  |  |
| L. pardinus-1 | Spain | 1 | L. canadensis-410 | Quebec | 2 |  |
| *L. pardinus-4* | Spain | 1 | *L. canadensis-901* | Maine | 2 |  |
| *L. pardinus-5* | Spain | 1 | *L. canadensis-904* | Maine | 2 |  |
| *L. pardinus-10* | Spain | 1 | *L. canadensis-905* | Maine | 2 |  |
| *L. pardinus-16* | Spain | 1 | *L. canadensis-908* | Maine | 2 |  |
| *L. pardinus-17* | Spain | 1 | *L. canadensis-909* | Maine | 2 |  |
| *L. pardinus-19* | Spain | 1 | *L. canadensis-41* | Newfoundland | 2 |  |
| *L. pardinus-20* | Spain | 1 | *L. canadensis-43* | Newfoundland | 2 |  |
|  |  |  | *L. canadensis-47* | Newfoundland | 2 |  |
| *L. lynx-12* | Switzerland | 2 | *L. canadensis-52* | Newfoundland | 2 |  |
| *L. lynx-29* | Belarus | 1 & 2 | *L. canadensis-53* | Newfoundland | 2 |  |
| *L. lynx-34* | Tibet | 2 | *L. canadensis-55* | Newfoundland | 2 |  |
| *L. lynx-35* | Xinijang | 2 | *L. canadensis-56* | Newfoundland | 2 |  |
| *L. lynx-36* | Xingxia | 1 & 2 | *L. canadensis-57* | Newfoundland | 2 |  |
| *L. lynx-37* | Xingxia | 2 | *L. canadensis-62* | Newfoundland | 2 |  |
| *L. lynx-38* | Xinijang | 1 | *L. canadensis-299* | Quebec | 2 |  |
| *L. lynx-39* | Xinijang | 2 | *L. canadensis-307* | Quebec | 2 |  |
| *L. lynx-40* | Gansu | 1 | *L. canadensis-308* | Quebec | 2 |  |
| *L. lynx-42* | Gansu | 2 | *L. canadensis-315* | Quebec | 2 |  |
| *L. lynx-43* | Gansu | 2 | *L. canadensis-318* | Quebec | 2 |  |
| *L. lynx-44* | Gansu | 1 & 2 | *L. canadensis-319* | Quebec | 2 |  |
| *L. lynx-45* | Ningxia | 1 & 2 | *L. canadensis-322* | Quebec | 2 |  |
| *L. lynx-46* | Ningxia | 1 |  |  |  |  |
| *L. lynx-47* | Qinghai | 2 |  |  |  |  |
| *L. lynx-48* | Qinghai | 2 |  |  |  |  |
| *L. lynx-49* | Qinghai | 1 |  |  |  |  |
| *L. lynx-50* | Qinghai | 1 & 2 |  | **Proportion of SINE Haplotypes** | | |
| *L. lynx-51* | Yunnan | 2 |  | **SINE 1** | **SINE 2** | |
| *L. lynx-53* | Russia | 2 | ***L. canadensis*** | **0%** | **100%** | |
| *L. lynx-54* | Russia | 2 | ***L. lynx*** | **17%** | **61%** | |
| *L. lynx-55* | Russia | 2 | ***L. pardinus*** | **100%** | **0%** | |
| *L. lynx-56* | Russia | 2 |  |  |  | |
