# Supplementary material for: The dynamic proliferation of CanSINEs mirrors the complex evolution of Feliforms
Source: BMC Evol Biol. 2014 Jun 20;14:137. doi: 10.1186/1471-2148-14-137 (PMC4084570; doi:10.1186/1471-2148-14-137)
Supplement: Additional file 5: Figure S1 — Four unique CanSINE insertion events at locus 133135. Alignment of four CanSINE insertion events occurring at locus 133135 in the caracal (Profelis caracal), marbled cat (Pardofelis marmorata), ocelot lineage (genus: Leopardus) and bobcat (Lynx rufus). The homologous region without CanSINE from F. catus sequence in included as a reference. The L. rufus CanSINE is in reverse orientation, RC (reverse complement). Yellow-highlighted regions mark target site duplications and gray-shaded regions denote the A and B RNA polymerase III recognition sequences. [file 1471-2148-14-137-S5.pdf]

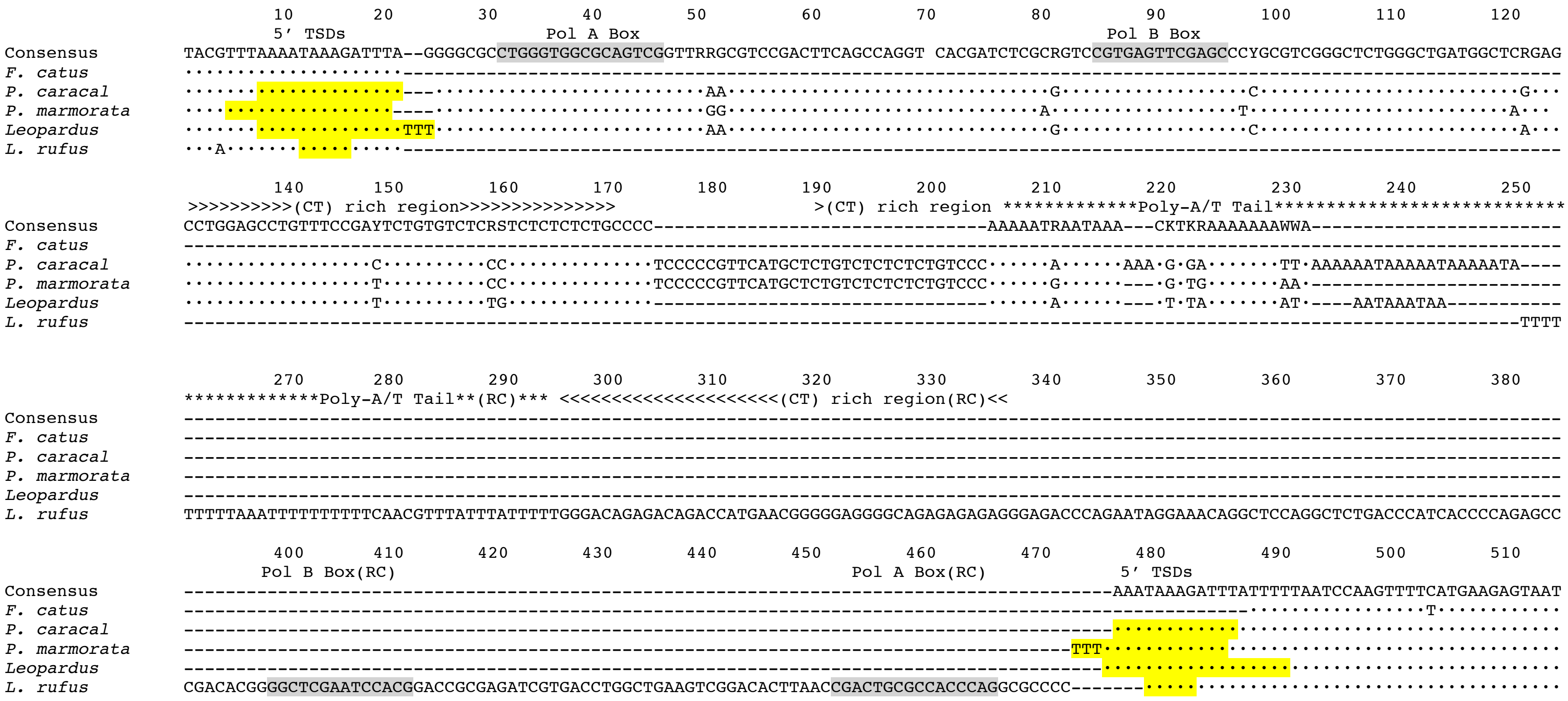

Figure S1 Alignment of four unique CanSINE insertion events occurring at locus 133135 in the caracal (*Caracal caracal*), marbled cat (*Pardofelis marmorata*), ocelot lineage (genus: *Leopardus*) and bobcat (*Lynx rufus*) with the homologous region without CanSINE from *F. catus* sequence as a reference. The *L. rufus* CanSINE is in reverse orientation, RC (reverse complement). Yellow-highlighted regions mark target site duplications and gray-shaded regions denote the A and B RNA polymerase III recognition sequences.
