# Supplementary material for: The dynamic proliferation of CanSINEs mirrors the complex evolution of Feliforms
Source: BMC Evol Biol. 2014 Jun 20;14:137. doi: 10.1186/1471-2148-14-137 (PMC4084570; doi:10.1186/1471-2148-14-137)
Supplement: Additional file 7: Figure S3 — Alignment of Lynx lineage individuals at locus 106256. Alignment of Lynx lineage individuals at locus 106256 reveals a conserved Can-SINE in all species, a Can-SINE immediately adjacent in L. pardinus and some L. lynx individuals, and a different Can-SINE insertion that begins 260 bp 3’ of the conserved SINE in all L. canadensis and some L. lynx individuals. Each insertion has unique TSDs, as well as distinct SNPs in the tRNA related region. Yellow-highlighted regions mark target site duplications and gray-shaded regions denote the A and B RNA polymerase III recognition sequences. [file 1471-2148-14-137-S7.pdf]

```
                                 10       20        30        40        50       60        70        80       90       100       110       120
                                  5’ TSD             Pol A Box                                               Pol B Box
Consensus               TCTCT-------------------------------------------------------------------------------------------------------------------------
S2 L.canadensis/L.lynx  ······························································································································
S1 L.pardinus/L.lynx    .....AAAATAAAAAATTAGGGGCGCCTGGGTGGCRCAGTCGGTTAAGCGTCCGACTTCAGCCAGGTCACTATCTCGCGGTCCGTGAGTTCGAGCCCCGCGTCGGGCTCTGGGCTGATGGCTCAGAG
S0 L.rufus              ······························································································································

                                 140     150       160       170       180       190       200       210       220       230      240       250
                                                  >>>>>>>>>CT Rich Region>>>>>>>>>>>>>>>>>>>>>>>>>>>*************Poly-A/T Tail**********************
Consensus               -------------------------------------------------------------------------------------------------------------------------------
S2 L.canadensis/L.lynx  ·······························································································································
S1 L.pardinus/L.lynx    CCTGGAGCCTGTTTCCGATTCTGTGTCTCCCTCTCTCTCTGCCCCTCCCCCGTTCATGCTCTGTCTCTCTCTGTCCCAAAAATAAATAAACGTTGAAAAAAAAAAATTTAAAAAAATAAAATAAAAT
S0 L.rufus              ·······························································································································

                                270      280       290      300       310       320       330       340       350       360       370        380
                           3’ TSD
Consensus               --------------ATTAATTAATTTTTAAAAAGTAGATGAGATTAGAAGAGGTGGAACCAAAATGGTGTAGGGACTTATAGACCTTGGCTTAACGTTTATATGTATATTTGTAAATTGTATATGTA
S2 L.canadensis/L.lynx  ·······························································································································
S1 L.pardinus/L.lynx    AAAATAAAAAATTA·················································································································
S0 L.rufus              ························································································································G······

                               400       410       420       430      440       450       460       470        480       490       500        510
Consensus               TATCTATTTCTTATGTGTATGTGTATATATGTACGTGTATCTGAAAAAGTAATGCTTCATAAAAAAATTTAGAAAGTACAAGTATAAAAGTTATGATCCTGCTGTCTTTACAAATAATAATTATTTAC
S2 L.canadensis/L.lynx  ································································································································
S1 L.pardinus/L.lynx    ·································T······························································································
S0 L.rufus              ································································································································

                               530       540      550      560       570       580        590        600        610        620       630       640
                                                                                                  Pol A Box
Consensus               ATTTTGATATAGGACCGCCAAGATTGTGTGT--CTCTGGGTGCATATTTNAAAAAAAAA-------------------------------------------------------------------
S2 L.canadensis/L.lynx  ·················································-AAAAAAAAACTACAGGGGCGCCTGGGTGGCGCAGTCGGTTAAGCGTCCGACTTCAGCCAGGTCACGATCTCGCGGT
S1 L.pardinus/L.lynx    ·······························TCT···············N····························································································
S0 L.rufus              ·················································A····························································································

                               660       680      690      700       710        720        730       740        750       760        770        780
                           Pol B Box                                                                    >>>>>>>>>>CT Rich Region>>>>>>>>>>>>        3’ TSD
Consensus               ------------------------------------------------------------------------------------------------------------------------------
S2 L.canadensis/L.lynx  CTGTGAGTTCGAGCCCCGCGTCGGGCTCTGGGCTGATGGCTCANAGCCTGGAGCCTGTTTCCGATTCTGTGTCTTCCTCTCTCTCTGCCCCTCCCCCGTTCATGCTCTGTCTCTCTCTGTCCCAAA
S1 L.pardinus/L.lynx    ······································································································----------------------------------------
S0 L.rufus              ······································································································----------------------------------------

                               790       800      810      820       830
Consensus               ---AAAAANNAAAAAAAAAAAAATACAGGGAAGGATCATATTACGTACTT
S2 L.canadensis/L.lynx  AAA······T·······································T
S1 L.pardinus/L.lynx    ---AAAAANNAAAAAAAAAA-C····························
S0 L.rufus              ---------------------·····························
```

Figure S3. Alignment of Lynx lineage individuals at locus 106256 reveals a conserved Can-SINE in all species, a Can-SINE immediately adjacent in *L. pardinus* and some *L. lynx* individuals, and a different Can-SINE insertion that begins 260 bp 3’ of the conserved SINE in all *L. canadensis* and some *L. lynx* individuals. Each insertion has unique TSDs, as well as distinct SNPs in the trna related region. Yellow-highlighted regions mark target site duplications and gray-shaded regions denote the A and B RNA polymerase III recognition sequences.
