# Supplementary material for: The dynamic proliferation of CanSINEs mirrors the complex evolution of Feliforms
Source: BMC Evol Biol. 2014 Jun 20;14:137. doi: 10.1186/1471-2148-14-137 (PMC4084570; doi:10.1186/1471-2148-14-137)
Supplement: Additional file 9: Figure S4 — Alignment of Lynx lineage individuals at locus 134463. Alignment of Lynx lineage individuals at locus 134463 reveals, a Can-SINE insert in L. canadensis (N = 7) and L. lynx (N = 3), yet absent from all 5 L. pardinus (N = 5). Yellow-highlighted regions mark target site duplications and gray-shaded regions denote the A and B RNA polymerase III recognition sequences. [file 1471-2148-14-137-S9.pdf]

```
                         10        20        30        40        50        60        70        80        90       100       110       120
                                           5’ TSD                    Pol A Box                                                       Pol B Box
Consensus        TGATGCTATATTAAAATGATTAGTAGAAAGCAGTCAAGAAAATGGGGGGCCTGGGTGGGGCAGTCGGTTAAGGGTCCGAATTCAGCCAGGTCACGATTTNGCGGTCCGTGAGTTCGAGCCCAGGGTCAGG
L. canadensis    ...................................................................................................T..............................
L. lynx          ...................................................................................................G..............................
L. pardinus      .......................-----------------------------------------------------------------------------------------------------------

                        140       150       160       170       180       190       200       210       220       230       240       250
                                                            >>>>>>>>>>CT Rich Region>>>>>>>>>>>>>>>>>>>>>>>*****************Poly AT tail*********
Consensus        GTTTGGGGTGATGGGTCAGAGCCTGGAGCCCGTTTTGATTTTGNGTTTCCCTTTTTTTTTGCCCCTCCCCCGTTCATGGTTTGTTTTTTTTTGTCCCAAAAATAAATAAACGTTGAAAAAAAAAAAATTT
L. canadensis    ...........................................G......................................................................................
L. lynx          ...........................................T......................................................................................
L. pardinus      ---------------------------------------------------------------------------------------------------------------------------------.

                        270       280       290       300
                        3’ TSD
Consensus        AGAAAGCAGTCAAGAAGAGATTCAGAAACGTAAGACACTT
L. canadensis    ........................................
L. lynx          ........................................
L. pardinus      ........................................
```

Figure S4. Alignment of lynx lineage individuals at locus 134463 reveals, a Can-SINE insert in all 7 *L. canadensis* and 3 *L. lynx* individuals examined, yet absent from all 5 *L. pardinus* individuals examined. Yellow-highlighted regions mark target site duplications and gray-shaded regions denote the A and B RNA polymerase III recognition sequences.
