# Supplementary material for: The dynamic proliferation of CanSINEs mirrors the complex evolution of Feliforms
Source: BMC Evol Biol. 2014 Jun 20;14:137. doi: 10.1186/1471-2148-14-137 (PMC4084570; doi:10.1186/1471-2148-14-137)
Supplement: Additional file 11: Figure S6 — Alignment of DNA sequences from locus 161275. Alignment of DNA sequences from locus 161275 SINE amongst Asian leopard cat lineage species, with NADH5 haplotypes for each individual indicated in parentheses. Yellow-highlighted regions mark target site duplications and gray-shaded regions denote the A and B RNA polymersase III recognition sequences. The 2 rusty-spotted cat (P. rubiginosa) individuals have SINE sequences that differ from each other and are found amongst the asian leopard cat (P. bengalensis). Thus it cannot be determined whether the rusty-spotted cats acquired their SINEs through incomplete lineage sorting of ancestral polymorphisms or through hybridization with the Asian Leopard cat. [file 1471-2148-14-137-S11.pdf]

```
                                     10        20        30        40        50        60        70        80        90       100       110       120
                                    5’TSD                Pol A Box                                             Pol B Box
P. viverrinus27,34,44(A,B,B) TGAACAATAAGGAAATACTGAATGTCCTTTTTAAGAACTGATTTGAGGGGCGCCTGGGTGGCGCAGTCGGTTAAGCGTCCGACTTCAGCCAGGTCACGATCTTGCGGTCCGTGAGTTCGA
P. viverrinus(38)            ........................................................................................................................
P. viverrinus(35)            ........................................................................................................................
P. viverrinus(20)            .................................................................................................A............A.........
P. viverrinus3(A)            ..................................A.....................................................................................
P. rubignosus2(C)            ..............................................................A.........................................................
P. rubignosus3(C)            .............................................................T.....................C....................................
P.bengalensis6(U)            .............................................................T.....................C....................................
P.bengalensis9(C)            ..............................................................A.........................................................
P.bengalensis18(B)           ..............................................................A.........................................................
P.bengalensis47,102,105(B)   ..............................................................A.........................................................

                                    130       140       150       160       170       180       190       200       210       220       230       240
                                                                                  >>>>>>>>>>CT Rich Region>>>>>>>>>**********Poly AT tail***********
P. viverrinus27,34,44(A,B,B) GCCCCACGTCGGGCCCTGGGCTGATGGCTCAGAGCCTGGAGCCTGTTTCCGATTCTGTGTCTCCCTCTCTCTCTGCCCTTCCCCCGTTCATGCTCTGTCTCTCTCTGTCCCAAAAATAAA
P. viverrinus38(A)           ........................................................................................................................
P. viverrinus35(A)           ........................................................................................................................
P. viverrinus20(A)           ........................................................................................................................
P. viverrinus3(A)            ........................................................................................................................
P. rubignosus2(C)            .....G..................................................................................................................
P. rubignosus3(C)            .....G.C................................................................................................................
P.bengalensis6(U)            .....G..................................................................................................................
P.bengalensis9(C)            .....G..................................................................................................................
P.bengalensis18(B)           .....G.........................R........................................................................................
P.bengalensis47,102,105(B)   .....G..................................................................................................................

                                    250         270       280       290       300       310
                             *********Poly AT tail********************  3’ TSD
P. viverrinus27,34,44(A,B,B) TAAACGTTGA-----------------AAACAAAAATTAAGAACTGATTTGAAATTAATTTCAATCCAT
P. viverrinus38(A)           ..........-----------------.............A..........................C.
P. viverrinus35(A)           ..........-----------------..............................G.....T.....
P. viverrinus20(A)           ..........-----------------..........................................
P. viverrinus3(A)            ..........-----------------..........................................
P. rubignosus2(C)            ..........-----------------..........................................
P. rubignosus3(C)            ..........AAAAAAAATTTTTTTTT...A..................-...................
P.bengalensis6(U)            ..........AAAAAAAATTTTTTTTT...A......................................
P.bengalensis9(C)            ..........-----------------..........................................
P.bengalensis18(B)           ..........-----------------.............A............................
P.bengalensis47,102,105(B)   ..........-----------------......................-...................
```

Figure S6 Alignment of DNA sequences from the scaffold 161275 SINE amongst asian leopard cat lineage species, with NADH5 haplotypes for each individual indicated in parentheses. Yellow-highlighted regions mark target site duplications and gray-shaded regions denote the A and B RNA polymersase III recognition sequences. The 2 rusty-spotted cat (P. rubiginosa) individuals have SINE sequences that differ from each other and are found amongst the asian Leopard cat (P. bengalensis). Thus it cannot be determined whether the rusty-spotted cats acquired their SINEs through incomplete lineage sorting of ancestral polymorphisms or through hybridization with the asian leopard cat.
