# Supplementary material for: The dynamic proliferation of CanSINEs mirrors the complex evolution of Feliforms
Source: BMC Evol Biol. 2014 Jun 20;14:137. doi: 10.1186/1471-2148-14-137 (PMC4084570; doi:10.1186/1471-2148-14-137)
Supplement: Additional file 13: Figure S8 — Alignment of a CanSINE insertion at locus 214534. Proliferation of the CanSINE at locus 214534 occurred during the initial Felidae radiation. Sequence from one species represents each lineage. The insertion is present in all lineages except the Panthera lineage. Within the caracal lineage, the insertion is unfixed within the african golden cat species but absent in multiple caracal and serval individuals. Yellow-highlighted regions mark target site duplications and gray-shaded regions denote the A and B RNA polymerase III recognition sequences. [file 1471-2148-14-137-S13.pdf]

```
                         10        20        30        40        50        60        70        80        90       100       110       120
                                                    5’TSD             Pol A Box                                                Pol B Box
Domestic       CACACTCACTTGAGAGCTCACTCAGTTATTGCTGCAAAAAATAGGGGCGCCTGGGTGGCTCAGTCGGTTAAGCGTCCGACTTTGGCTCAGGTCATGATCTCGCGGTATGTGAGTTCGAGCCCTGCGTCGG
Asian_Leopard  ............................................................................T.................C........T..........................
Lynx           ..........................................................................G.......C...........C...AC......................CA......
Puma           ...................G...........T......................A...G......-.......TG.T.....C..A........C....................T......C.......
Ocelot         ...................G.....................................C........................C...........C...........................C....T..
Caracal        ...................G...............-----------------------------------------------------------------------------------------------
Bay            ...................G..........................................................G...............C...........................C....T.A
Panthera       ..................TG...............-----------------------------------------------------------------------------------------------

                         140       150       160       170       180       190       200       210       220       230       240       250
                                                           >>>>>>>CT Rich Region>>>>>>>>>>>>>>>>>>>>>>>>>>>>>>>>>**Poly AT tail************
Domestic       GCTCTGTGCTGACTGCTCAGAGCCTGGAGTCTGTTTCAGATTCTGTGTCTCCCTCTCTCTCTGACCCTCTCCCGTTCATGCTCTGTCTCTCTCTGTCTCAA----AAATAAATGTTAAAAAAAA-TAAAA
Asian_Leopard  .............................C.......................................................................----...........-.......T.....
Lynx           .............................C.................................................------................AAAT...................-.....
Puma           .............................C..........................M.A.Y...............................S........AAAT...........T.......T.....
Ocelot         .............................C................................T..........A.....T.....................AAAT...................T.....
Caracal        -------------------------------------------------------------------------------------------------------------------------------...
Bay            ..--.........................C.......................................................................AAAT...................A.....
Panthera       -------------------------------------------------------------------------------------------------------------------------------...

                         270       280       290       300       310       320
               3’TSD
Domestic       AAATAGATATTTATTTATTGATGCCTAGAAGTTATCCCAAAATTCAATAGTCTAAAACGACCGTTATATA
Asian_Leopard  ..............................A...........C.T.G........M.....T........
Lynx           ..........................................C.T.G..............T........
Puma           ...............----.......................C.T.GW.............T........
Ocelot         ...............----.......................C.T.G..............T........
Caracal        .................................G........C.T.G..............T........
Bay            ..........................................C.T.G..............T........
Panthera       ................................G.........C.T.G............G.T........
```

Figure S8 Alignment of a SINE insertion at locus 214534, which occurred during the initial Felidae radiation. Sequence from one species represents each lineage. The insertion is present in all lineages except the Panthera lineage. Within the Caracal lineage, the insertion is unfixed within the African Golden Cat species but absent in multiple Caracal and Serval individuals. Yellow-highlighted regions mark target site duplications and gray-shaded regions denote the A and B RNA polymerase III recognition sequences.
