# Supplementary material for: The dynamic proliferation of CanSINEs mirrors the complex evolution of Feliforms
Source: BMC Evol Biol. 2014 Jun 20;14:137. doi: 10.1186/1471-2148-14-137 (PMC4084570; doi:10.1186/1471-2148-14-137)
Supplement: Additional file 10: Figure S5 — Alignment of ocelot (Leopardus) lineage individuals at locus 133135. Alignment of ocelot (Leopardus) lineage individuals at locus 133135 reveals a Can-SINE insert in L. pardalis(N = 9) L. tigrina (N = 8), O. geoffroyi (N = 8) and O. guigna (N = 1) and L.jacobita (N = 1) individuals, yet absent from L. colocolo (N = 3) and L. wiedii (N = 2). Yellow-highlighted regions mark target site duplications and gray-shaded regions denote the A and B RNA polymerase III recognition sequences. [file 1471-2148-14-137-S10.pdf]

```
                          10       20      30         40      50      60        70        80      90      100      110      120
                                                5’TSD               Pol A Box                                             Pol B Box
Consensus        AAACTCTGGTTATACGTTTAAAATAAAGATTTATTTGGGGCGCCTGGGTGGCGCAGTCGGTTAAGCGTCCGACTTCAGCCAGGTCACAATCTCGCGGTCCGTGAGTTCGAGCCCCGCGTCGGGCTCTGGG
F.catus          ....................................----------------------------------------------------------------------------------------------
L.colocolo       ....................................----------------------------------------------------------------------------------------------
L.wiedii         ....................................----------------------------------------------------------------------------------------------
O.guigna         ..................................................................................................................................
L.pardalis       ..................................................................................................................................
L.jacobita       ..................................................................................................................................
L.tigrina        ..................................................................................................................................
O.geoffroyi      ..................................................................................................................................

                          140      150       160       170       180       190       200       210       220       230      240       250
                                          >>>>>>>>>CT Rich Region>>>>>>>>>>>>>*****************Poly AT tail*******   3’ TSD
Consensus        CTGATGGCTCAGAGCCTGGAGCCTGTTTCCGATTCTGTGTCTCTGTCTCTCTCTGTCCCAAAAATAAATAAACTTTAAAAAAAAATAAATAAATAACAAATAAAGATTTATTTTTAATCCAAGTTTTCAT
F.catus          -----------------------------------------------------------------------------------------------------------------.................
L.colocolo       -----------------------------------------------------------------------------------------------------------------.................
L.wiedii         -----------------------------------------------------------------------------------------------------------------.................
O.guigna         ..................................................................................................................................
L.pardalis       ..................................................................................................................................
L.jacobita       .....................................................................................--------.....................................
L.tigrina        ..................................................................................................................................
O.geoffroyi      ..................................................................................................................................
```

Figure S5. Alignment of Ocelot (*Leopardus)* lineage individuals at locus 133135 reveals, a Can-SINE insert in all 9 *L. pardalis*, 8 *L. tigrina,* 8 *O. geoffroyi* and each *O. guigna* and *L.jacobita* individuals examined, yet absent from all 3 *L. colocolo* and 2 *L. wiedii* individuals examined. Yellow-highlighted regions mark target site duplications and gray-shaded regions denote the A and B RNA polymerase III recognition sequences.
