# Supplementary material for: The dynamic proliferation of CanSINEs mirrors the complex evolution of Feliforms
Source: BMC Evol Biol. 2014 Jun 20;14:137. doi: 10.1186/1471-2148-14-137 (PMC4084570; doi:10.1186/1471-2148-14-137)
Supplement: Additional file 12: Figure S7 — Alignment of a CanSINE insertion at locus 154966. Proliferation of the CanSINE at locus 154966 occurred during the initial Felidae radiation. Sequence from one species represents each lineage. The insertion is present in the domestic cat, asian leopard cat and lynx lineages. Yellow-highlighted regions mark target site duplications and gray-shaded regions denote the A and B RNA polymerase III recognition sequences. [file 1471-2148-14-137-S12.pdf]

```
                        10        20        30        40        50       60        70        80       90       100       110       120
                                                      5’TSD                Pol A Box                                              Pol B Box
Domestic       TCCTTCCAATTTCCTATGTACTTTATGTTCGTTGTAGAAAATGTGGTAGAGGGGCGCCTGGGTGGCGCAGTCGGTTAAGCGTCCGACTTCAGCCAGGTCACGATCTCGCGGTCCGTGAGTTCGAGCCCCG
Asian_Leopard  ..................................................................A................................................G.............A
Puma           ...................................-----------------------------------------------------------------------------------------------
Lynx           ..................................................................A...............................................................
Ocelot         ..............................A....-----------------------------------------------------------------------------------------------
Caracal        .....M.............................-----------------------------------------------------------------------------------------------
Bay            ...................................-----------------------------------------------------------------------------------------------
Panthera       ...................................-----------------------------------------------------------------------------------------------

                       140        150       160       170       180       190       200       210       220       230      240       250

                                                    >>>>>>>>>>>>>>>>>>>>CT Rich Region>>>>>>>>>>>>>>>>>>>>>>>>>>>>>>>>>>>>>>****Poly AT tail*****
Domestic       CGTCGGGCTCTGGGCTGATGGCTCGGAGCCTGGAGCCTGTTTCCGATTCTGTGTCTCCC--TCTCTCTCTGCCCCTCCCCCGTTCATGCTCTGTCTCTCTCTGTCCCTAAAATAAATAAAAGTTGAAAAA
Asian_Leopard  ....A...................A..........A.......................TC.........................G....................A......................
Puma           ----------------------------------------------------------------------------------------------------------------------------------
Lynx           ..................................A........................--..............................................C......................
Ocelot         ----------------------------------------------------------------------------------------------------------------------------------
Caracal        ----------------------------------------------------------------------------------------------------------------------------------
Bay            ----------------------------------------------------------------------------------------------------------------------------------
Panthera       ----------------------------------------------------------------------------------------------------------------------------------

                        270       280       290       300       310       320
               ***************    3’ TSD
Domestic       AAAATTTTTTTTTAAAGAAAATGTGGTAGATATAAACAAAGAAGGGAACATCACCCCGAATCCCATGACT
Asian_Leopard  ...........-A...........................................T........C....
Puma           ---------------..................................................CA...
Lynx           ...........-A....................................................C....
Ocelot         ---------------................G......................................
Caracal        ---------------..................................................CR...
Bay            ---------------..................................................C....
Panthera       ---------------.......................................................
```

Figure S7 Alignment of a SINE insertion at locus 154966, which occurred during the initial Felidae radiation. Sequence from one species represents each lineage. The insertion is present in the Domestic Cat, Asian Leopard Cat and Lynx lineages. Yellow-highlighted regions mark target site duplications and gray-shaded regions denote the A and B RNA polymerase III recognition sequences.
